# Microsporidian Infection in Mosquitoes (Culicidae) Is Associated with Gut Microbiome Composition and Predicted Gut Microbiome Functional Content

**DOI:** 10.1007/s00248-021-01944-z

**Published:** 2021-12-23

**Authors:** Artur Trzebny, Anna Slodkowicz-Kowalska, Johanna Björkroth, Miroslawa Dabert

**Affiliations:** 1 Molecular Biology Techniques Laboratory, Faculty of Biology, Adam Mickiewicz University, Poznan, Poland; 2 Department of Biology and Medical Parasitology, Poznan University of Medical Sciences, Poznan, Poland; 3 Department of Food Hygiene and Environmental Health, Faculty of Veterinary Medicine, University of Helsinki, Helsinki, Finland

**Keywords:** Indicator taxon analysis, Metagenome functional content, Microsporidia, Mosquito gut microbiota, Microsporidian infection, PICRUSt2, *Spiroplasma*, *Weissella*

## Abstract

The animal gut microbiota consist of many different microorganisms, mainly bacteria, but archaea, fungi, protozoans, and viruses may also be present. This complex and dynamic community of microorganisms may change during parasitic infection. In the present study, we investigated the effect of the presence of microsporidians on the composition of the mosquito gut microbiota and linked some microbiome taxa and functionalities to infections caused by these parasites. We characterised bacterial communities of 188 mosquito females, of which 108 were positive for microsporidian DNA. To assess how bacterial communities change during microsporidian infection, microbiome structures were identified using 16S rRNA microbial profiling. In total, we identified 46 families and four higher taxa, of which *Comamonadaceae*, *Enterobacteriaceae*, *Flavobacteriaceae* and *Pseudomonadaceae* were the most abundant mosquito-associated bacterial families. Our data suggest that the mosquito gut microbial composition varies among host species. In addition, we found a correlation between the microbiome composition and the presence of microsporidians. The prediction of metagenome functional content from the 16S rRNA gene sequencing suggests that microsporidian infection is characterised by some bacterial species capable of specific metabolic functions, especially the biosynthesis of ansamycins and vancomycin antibiotics and the pentose phosphate pathway. Moreover, we detected a positive correlation between the presence of microsporidian DNA and bacteria belonging to *Spiroplasmataceae* and *Leuconostocaceae*, each represented by a single species, *Spiroplasma* sp. PL03 and *Weissella* cf. *viridescens,* respectively*.* Additionally, *W.* cf. *viridescens* was observed only in microsporidian-infected mosquitoes. More extensive research, including intensive and varied host sampling, as well as determination of metabolic activities based on quantitative methods, should be carried out to confirm our results.

**Supplementary Information:**

The online version contains supplementary material available at 10.1007/s00248-021-01944-z.

## Introduction

Microsporidians (Microsporidia) are among the most widespread, obligate intracellular parasites, causing mainly zoonotic or waterborne infections in humans, but able to infect almost all animal phyla [1]. Among 1700 described microsporidian species [2], more than 700 were isolated from insects [3], with mosquitoes (Culicidae) the most common [4, 5]. It is noted that microsporidians represent one of the largest and most diverse groups of parasites associated with mosquito populations in nature [4].

Mosquitoes can act as vectors for many disease-causing viruses and parasites and even carry and transmit multiple pathogens in a single host, creating numerous opportunities for interactions among vertebrate hosts, vectors, and pathogenic organisms. These interactions can occur on multiple levels and may ultimately affect transmission patterns and disease pathogenesis. One example is the possible immunosuppressive effect of filarial nematodes on malaria leading to reduced *Plasmodium* infectivity in mosquitoes [6]. The lowering of the efficiency of the infection of viral diseases, such as dengue or Zika, was also recorded in the co-occurrence of viruses and entomopathogenic fungi [7] or the bacterial endosymbiont *Wolbachia* spp. [8]. Moreover, there are reports that the insect microbiome can modulate the vector competence for arboviruses in *Aedes* and *Culex* mosquitoes [9].

The effects of microsporidians on the development of disease-causing organisms in mosquitoes have been studied mainly for malaria parasites for developing novel strategies to control mosquito populations or their capability to transmit *Plasmodium* parasites [10–12]. It has been shown that *Vavraia culicis* impairs the development of *Plasmodium berghei* by priming the immune melanisation response in the adult mosquito [11]. However, in mosquitoes, like in other insects, microsporidians also may affect the functions [13] and behaviour of the host, such as decreased lifespan, oviposition [14] or flight activity [15]. Interestingly, some of the mechanisms underlying these changes involve alterations in commensal bacterial species in the host gut microbiome [16, 17]. A recent study showed that infection of *Anophele*s *arabiensis* with a vertically transmitted microsporidian species prevented *P. falciparum* transmission because it reduced the establishment of *Plasmodium* oocysts in the *Anopheles* midgut and impaired the colonisation of *Anopheles* salivary glands by *Plasmodium* sporozoites [10]. Moreover, it has been shown that *Nosema ceranae* is to some extent associated with changes in gut microbiome structure and its presence is strongly correlated with some gut microbiome members (e.g. *Gilliamella* spp. in the honey bee, *Apis mellifera*) [18].

It has been increasingly recognised that microorganisms may participate in host–parasite interactions [19]. The gut microbiome may also have important direct and indirect effects on parasite establishment, including insect hosts [20, 21]. Recently, a proof-of-concept study showed significant changes in the gut bacteriome and mycobiome of the grain beetle (*Tenebrio molitor*) in association with tapeworm (*Hymenolepis diminuta*) infection [22]. Like that of other insects, the gut microbiome of mosquitoes mediates the interactions between the host and intestinal parasites by stimulating the host immune responses [23, 24] or protecting the host by inhibiting parasites, for example [25, 26]. These interactions have a promising application to prevent pathogen transmission because they can affect the symbiont populations and shape the microbial community structure of the host [27, 28].

Many studies have shown that sex, stage of development, host environment, diet type and pathogenic infection can influence the mosquito microbiota [26, 29–37]. Mosquito larvae live in water and so acquire the majority of their gut microbiota from their environment. Therefore, high water temperature, pH and oxygen content, and an abundance of residual antibiotics in breeding sites of mosquito larvae shape their microbiome [30–33]. During metamorphosis, the larval midgut bacteria are largely eliminated because of meconium egestion by the newly hatched adult mosquito [26, 29, 34]. Therefore, in adult mosquitoes, the most important factors determining microbiota are the source of blood meal, blood digestion and nectar assimilation or variations in mosquito sex and size [35–37].

Mosquito–microbiome interactions play an important role in mosquito biology, including the development of pathogens [38, 39]. Symbiotic bacteria affect the development of pathogens via the production of metabolites or by stimulating the host immunological responses [38, 40]. Additionally, it has been shown that the pathogenic bacterium *Serratia marcescens* as a microbiome member may enhance dengue virus infection [41] or inhibit *P. berghei* infection [28]. It was also demonstrated that *Wolbachia* spp. may repress [42] or inhibit *P. falciparum* [43] in *Anopheles gambiae*. Although mosquitoes are common hosts of microsporidia, there is a lack of data on their effects on the mosquito microbiota. Therefore, the present study aimed to detect changes in mosquito gut microbiota associated with microsporidian infection. As a model, we used mosquitoes collected from natural populations.

## Methods

### Material

For this study, we used 188 DNA isolates extracted from 188 female mosquitoes collected for a previous study [44] between July and August 2016 from the periphery of mixed birch–oak and riparian forests near the city of Poznan, western Poland. Here, we used representatives of five species: *Aedes vexans* (19), *Coquillettidia richiardii* (16), *Ochlerotatus annulipes* (63), *O*. *cantans* (77) and *O*. *sticticus* (13). Among them, 108/188 (58%) were positive for one or more microsporidian species (Table 1). Infection with Microsporidium sp. PL01 predominated in all tested mosquito species. Negative control samples from blank extractions, including solution used to wash mosquitoes before DNA extraction, were analysed in the same way as the test samples.

**Table 1:** DNA isolates from microsporidian-infected and non-infected mosquitoes used in this study. Higher numbers of infected mosquitoes result from co-infection events with more than one microsporidian species

Microsporidian species	Mosquito species (infection/individuals)					
	*Ae. vexans* (13/19)	*C. richiardii* (9/16)	*O. annulipes* (34/63)	*O. cantans* (44/77)	*O. sticticus* (8/13)	All mosquitoes (108/188)
*Amblyospora salinaria*					1	1
*Amblyospora* sp.			1	1	1	3
*Encephalitozoon hellem*				2		2
*Enterocytospora artemiae*			1			1
Microsporidium sp. nov. PL01	10	9	31	27	7	84
*Nosema adaliae*			1			1
*N. ceranae*				2		2
*N. chrysorrhoeae/portugal*	2	1	4	16		23
*N. pieriae*		1	2			3
*N. thomsoni*	1					1
*Nosema* sp. CHW-2007a	2	1	2	1	1	7

### Library Construction and NGS Sequencing

For 16S rRNA microbial profiling, we used V4F (CGATCAGCAGCCGCGGTAATA) and V4R (ATGGACTACCAGGGTATCTAA) primers targeting the V4 region in prokaryotic 16S rRNA gene [45]. Primers were tailed at 5'-ends with dual-indexed Ion Torrent adapters for sequencing using the Ion Torrent system (Life Technologies, USA). PCR reactions were done in two technical replications, each in a total volume of 10 µl, containing Hot FIREPol DNA Polymerase (Solis BioDyne, Estonia), 0.25 µM of each primer and 1 µl of template DNA. The PCR program was as follows: 95 °C for 12 min, followed by 30 cycles at 95 °C for 15 s, 50 °C for 1 min and 72 °C for 45 s, with a final extension step at 72 °C for 5 min. After PCR, technical replications were pooled and, for each sample, 3 µl was separated by electrophoresis on a 2% agarose gel to check amplification efficiency. Then, all samples were pooled in equal quantities and then purified using the 2% E-Gel SizeSelect II Agarose Gels system (Invitrogen, USA), according to the manufacturer’s instructions.

DNA concentration and fragment length distribution of the library were established using the High Sensitivity D1000 Screen Tape assay on a 2200 Tape Station system (Agilent, USA). Clonal template amplifications were performed using the Ion Torrent One Touch System II and the Ion Torrent OT2 Kit (Life Technologies) according to the manufacturer’s instructions. Sequencing was carried out using the Ion 540 Kit-OT2 and Ion Torrent S5 system on the Ion 540 chip (Life Technologies) according to the manufacturer’s instructions.

### Read Processing and Data Analysis

Raw sequence data were pre-filtered using the Ion Torrent Suite software version 5.10.1 (Life Technologies) to remove polyclonal and low-quality sequences. Further bioinformatic analyses were conducted using fastq data and custom workflow. Sequence reads shorter than 200 bp were removed from the dataset using Geneious R11.1.5 (Biomatters Ltd.). Leading and trailing low-quality bases were removed using Trimmomatic version 0.39 [46]. FASTX-Toolkit [47] was used to extract sequences with a minimum of 50% bases with a quality score of ≥ 25. Quality-filtered sequences were separated by barcodes and trimmed at 5'- and 3’-ends to exclude PCR primers in Geneious R11.1.5. The singletons (< 10 reads) were removed using the fastx_uniques and sortbysize algorithms [48]. Chimeras were removed using the default settings in UCHIME2 version 4.2.40 [49].

Operational taxonomic unit (OTU) clustering at 97% similarity was done in USEARCH version 11.0.667 [48]. Sequences were denoised into zero-radius operational taxonomic units (ZOTUs), and subsequently, a ZOTU table was constructed according to the denoising steps [49]. The ZOTU table was then corrected for the 16S copy number based on the unbias algorithm. Phylogenetic affiliations were analysed by the usearch sintax algorithm using a confidence threshold of 0.8 [50–53]. ZOTUs were compared against the SILVA database for ARB for small subunit ribosomal RNAs version 138 [54–56]. ZOTUs detected in control samples were used to identify cross-talk errors among the analysed mosquito samples. The UNCROSS2 algorithm was used to remove ZOTUs detected in control samples from the dataset [57]. Then, the reads were normalised by the otutab_rare algorithm [51] to compare sample diversities.

The functional potential of prokaryotic communities in all samples was predicted using the Phylogenetic Investigation of Communities by Reconstruction of Unobserved States (PICRUSt2) version 2.4.1 software package [58]. The ZOTU table normalised by 16S rRNA gene copy number was used for metagenome functional prediction, generating a table of Kyoto Encyclopedia of Genes and Genomes (KEGG) Orthologs (KOs) [59–61]. The predictions were categorised at KEGG Orthology level 1, 2 and 3 within the pathway hierarchy of KEGG. As an indicator for the PICRUSt2 prediction accuracy, the Nearest Sequenced Taxon Index (NSTI) for each sample was estimated and calculated [62]. The comparison of potential functions among different sample categories was supplemented by the Statistical Analysis of Metagenomic Profiles (STAMP) version 2.1.3 [63].

### Amplification of Complete 16S rRNA Gene from Weissella

To confirm the taxonomic position of the *Weissella* identified by NGS, a near-complete *Weissella* 16S rRNA gene was amplified using genus-specific S-G-Wei-0121-a-S-20 (CGTGGGAAACCTACCTCTTA) and S-G-Wei-0823-a-A-18 (CCCTCAAACATCTAGCAC) primers [64]. PCR reactions were prepared in two technical replicates, each in a total volume of 10 µl, containing Hot FIREPol DNA Polymerase, 0.25 µM of each primer and 1 µl of template DNA. The amplification program was as follows: 95 °C for 12 min, followed by 35 cycles at 95 °C for 15 s, 60 °C for 1 min and 72 °C for 1 min, with a final extension step at 72 °C for 10 min. After amplification, technical replications were pooled, and 5 μl was analysed by electrophoresis on 1.5% agarose gel.

Samples containing visible bands were purified with *Escherichia coli* exonuclease I and FastAP Alkaline Phosphatase (Thermo Scientific, USA) and sequenced using the BigDye v3.1 Kit and ABI Prism 3130XL Genetic Analyzer (Applied Biosystems, USA), following the manufacturer’s instructions. Sequence chromatograms were checked for accuracy in Geneious R11.1.5. Then, sequences were compared to GenBank using blastn [65] optimized for highly similar sequences (megablast algorithm) [66].

### Spiroplasma Identification Using Phylogenetic Analysis

To identify the *Spiroplasma* species isolated from mosquitoes, all 16S sequences assigned to the genus *Spiroplasma* published in GenBank (www.ncbi.nlm.nih.gov) were used. As close outgroups, the 16S sequences of *Clostridium ramosum* and five *Mycoplasma* spp. were used. The bacterial strains and GenBank accession numbers for DNA sequences used in the *Spiroplasma* spp. phylogenetic analysis are provided in Supplementary Table 1.

Sequences were aligned using the L–INS–I algorithm in MAFFT version 7.388 [67], as implemented in Geneious R11.1.5. The best-fit model of DNA evolution (GTR + I + G) was chosen by PartitionFinder2 [68]. Phylogenetic trees were reconstructed using maximum likelihood (ML) in Garli version 2.0 [69] and Bayesian inference (BI) in MrBayes version 3.2.6 [70]. Each BI run of four independent chains was performed in 4 × 10,000,000 generations, and the trees were sampled every 1000 generations. The final consensus tree was generated after discarding the burn-in fraction of 0.25% of initial trees; the average standard deviation of split frequencies dropped below 0.002. Bootstrap support for the ML tree was calculated by using 1000 data replicates as implemented in Garli. The trees were edited in FigTree version 1.4.4 [71] and further edited in Corel Draw X4.

### Statistical Analyses

The Chao1 index and Shannon diversity in individual samples were calculated using the alpha_div algorithm [51]. Indexes were analysed using one-way ANOVA. The remaining statistical analyses were performed using the R software version 4.0.2 [72]. Visualization of the heatmap and cluster tree based on unweighted pair-group method with arithmetic median (UPGMA) were conducted in STAMP version 2.1.3 [63]. Statistical analyses were performed using permutation tests implemented in the coin package version 1.4–1 [73]. Comparisons between two independent groups were conducted using the Wilcoxon–Mann–Whitney (WMW) test. Comparisons of more than two groups were conducted using the Kruskal–Wallis test. To assess the difference between two independent groups, a statistic *r* was used, defined as *r* =|*Z*| / √N, where “*Z*” is the WMW test statistic and “N” is the number of observations. The relationship between the two variables was tested using the Spearman correlation coefficient (rho, *ρ*).

The correlation between microbiota composition and mosquito species, in addition to the effects of microsporidian infection and their interaction with mosquito microbiome composition, was tested using the analysis of similarities (ANOSIM) with 999 permutations [74]. Variation in microbial community composition and pathways’ structure differentiation was visualised using principal coordinate analysis (PCoA). An indicator species analysis was performed to determine whether taxa were exclusively found in infected or non-infected groups and if these taxa were commonly found in certain treatment groups, as revealed by the A and B components of the indicator species analysis, respectively. The indicator species analysis was performed using the multipatt function within the indicspecies package [75].

Comparisons of the two groups and correlation analysis were tested considering mosquitoes belonging to different species using stratified permutation tests. Additionally, statistical significances among *Weissella* and mosquito species were calculated based on the chi-square test. Any *p*-values of ≤ 0.05 were considered significant.

## Results

### Bacterial Communities in Field‑Collected Mosquitoes

After quality-filtering the samples, 18,006,087 reads were yielded. The average read number per sample was 95,777 (SD = 9 343). The clustering of ZOTUs across samples produced 1437 unique ZOTUs. The median of ZOTUs was 96 and ranged between 84 and 112. There were no differences in species richness (Chao1 index; F = 1.67; *p* > 0.848; Supplementary Fig. 1) and diversity (Shannon diversity; F = 1.77; *p* > 0.758; Supplementary Fig. 2) between microsporidia-positive and non-infected mosquitoes belonging to different species.

**Fig. 1:**
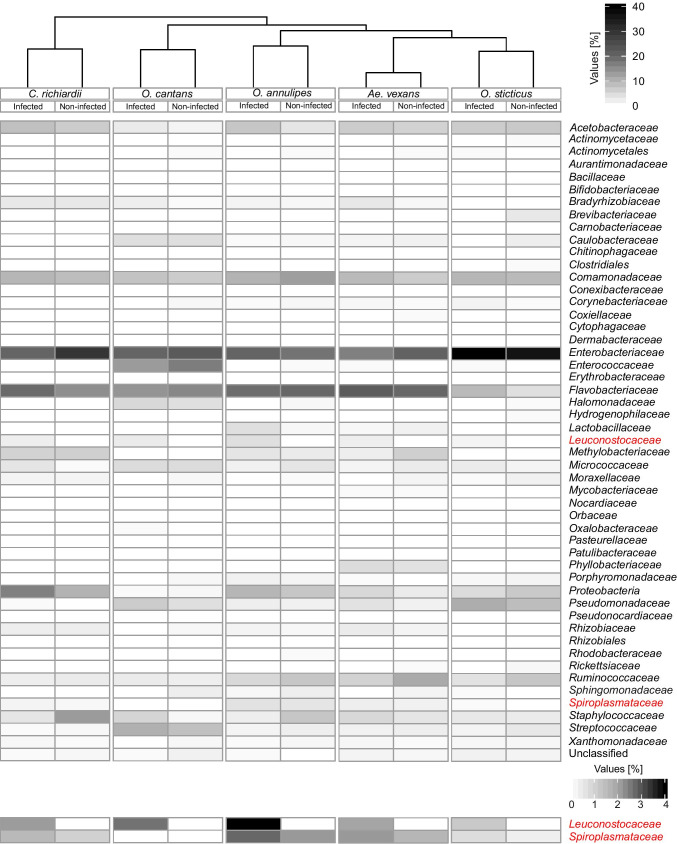
Heatmap of hierarchical clustering of family-level bacterial microbiome composition profiles. Darker colour represents higher abundance in mosquitoes' gut microbiota. ZOTUs not assigned to families were grouped into higher taxa

ZOTUs were clustered into 46 families and four higher taxa (Fig. 1). Proteobacteria (59%; SD = 12), Bacteroidetes (19%; SD = 11) and Firmicutes (17%; SD = 8) were the most highly abundant phyla associated with mosquitoes tested in this study (Supplementary Fig. 3, Supplementary Table 2). Among them, for both infected and non-infected mosquitoes, the most dominant families were *Enterobacteriaceae* (infected 19%, SD = 10; non-infected 20%; SD = 11), *Flavobacteriaceae* (infected 17%, SD = 11; non-infected 18%; SD = 11) and *Comamonadaceae* (infected 8%, SD = 6; non-infected 10%; SD = 6). Diversity analysis of mosquito microbiota based on the cluster tree using UPGMA showed that the diversity of bacterial communities was determined by mosquito species rather than microsporidian infection (Fig. 1).

**Fig. 2:**
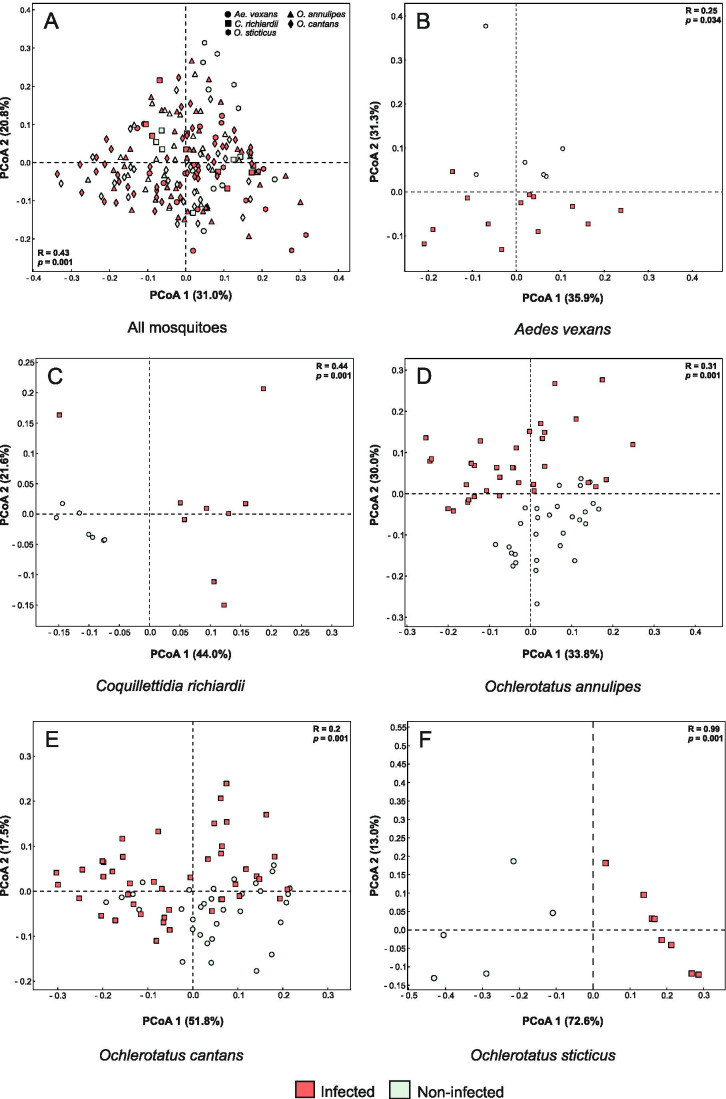
Gut microbiome structure differentiation and inter-individual similarity based on identified bacterial species between (**A**) all mosquitoes, (**B**) *Aedes vexans*, (**C**) *Coquillettidia richiardii*, (**D**) *Ochlerotatus annulipes*, (**E**) *O. cantans* and (**F**) *O. sticticus*. Mosquito species are categorised as infected (red squares) or non-infected (green circles). The correlation between microbiota composition and microsporidian infection (**B**–**F**) and between microbiota composition and both mosquito species and microsporidian infection (**A**) was tested using the analysis of similarities (ANOSIM) with 999 permutations. R value indicates the ANOSIM statistic which compares the mean of ranked dissimilarities between groups to the mean of ranked dissimilarities within groups, while *p*-value is statistically significant

Unlike the PCoA results of all mosquito microbiota as a pooled sample (i.e. without separation by host species), which did not group the bacterial communities according to microsporidian infection (Fig. 2A), the ANOSIM significantly differentiated them as associated with this infection (*R* = 0.09, *p* < 0.001) and mosquito species (*R* = 0.43, *p* < 0.001). By contrast, the results of PCoA for separate host species showed that the occurrence of microsporidians clearly differentiated the observed microbiome compositions (Fig. 2B − F).

Indicator species analysis for infected and non-infected mosquitoes (Table 2) showed that among the non-infected individuals, the families *Aurantimonadaceae*, *Conexibacteraceae*, *Cytophagaceae*, *Dermabacteraceae* and *Hydrogenophilaceae* displayed high values for the exclusive taxon component (A > 0.9). Additionally, the *Actinomycetaceae*, *Halomonadaceae*, *Methylobacteriaceae*, *Moraxellaceae*, *Mycobacteriaceae* and *Sphingomonadaceae* were noticed in all mosquitoes without microsporidians (B = 1). Mosquitoes infected with microsporidians were characterised by the exclusive occurrence of the *Leuconostocaceae* (A = 1). Moreover, the *Acetobacteraceae*, *Bradyrhizobiaceae*, *Pseudomonadaceae* and *Rhizobiaceae* were noticed in all infected mosquitoes (B = 1), while the *Spiroplasmataceae* was associated with the presence of microsporidians due to a high B-component value (B > 0.9).

**Table 2:** Indicator taxon analysis for infected and non-infected mosquitoes. Bacterial families that are significant indicators of community composition are shown. The “A component” indicates how exclusive the family was for infected or non-infected mosquitoes, where a value of 1 indicates that the family was exclusively found in that group. The “B component” indicates how frequently the given family was found in replicate samples, where 1 means it was found in every sample. The indicator value accounts for both the A and B components together

Infection	Family	A component	B component	Indicator value	*p*-value
Non-infected	*Actinomycetaceae*	0.633	1	0.795	0.010
	*Aurantimonadaceae*	0.933	0.838	0.884	0.005
	*Bacillaceae*	0.733	0.913	0.818	0.005
	*Brevibacteriaceae*	0.480	0.800	0.620	0.025
	*Conexibacteraceae*	0.934	0.538	0.708	0.010
	*Coxiellaceae*	0.680	0.925	0.793	0.005
	*Cytophagaceae*	0.932	0.125	0.341	0.030
	*Dermabacteraceae*	0.936	0.450	0.649	0.005
	*Erythrobacteraceae*	0.885	0.400	0.595	0.010
	*Halomonadaceae*	0.854	1	0.924	0.005
	*Hydrogenophilaceae*	0.901	0.888	0.894	0.005
	*Methylobacteriaceae*	0.588	1	0.767	0.015
	*Moraxellaceae*	0.682	1	0.826	0.005
	*Mycobacteriaceae*	0.597	1	0.773	0.025
	*Orbaceae*	0.606	0.850	0.718	0.005
	*Pseudonocardiaceae*	0.561	0.913	0.715	0.015
	*Rhodobacteraceae*	0.825	0.775	0.799	0.005
	*Rickettsiaceae*	0.853	0.825	0.839	0.005
	*Ruminococcaceae*	0.607	0.938	0.754	0.005
	*Sphingomonadaceae*	0.742	1	0.861	0.005
Infected	*Acetobacteraceae*	0.610	1	0.781	0.005
	*Bradyrhizobiaceae*	0.629	1	0.793	0.005
	*Leuconostocaceae*	1	0.139	0.373	0.005
	*Patulibacteraceae*	0.640	0.843	0.734	0.020
	*Pseudomonadaceae*	0.609	1	0.780	0.010
	*Rhizobiaceae*	0.623	1	0.789	0.005
	*Spiroplasmataceae*	0.616	0.926	0.755	0.005

### Gut Microbiome Members Associated with Microsporidian Infection

**Fig. 3:**
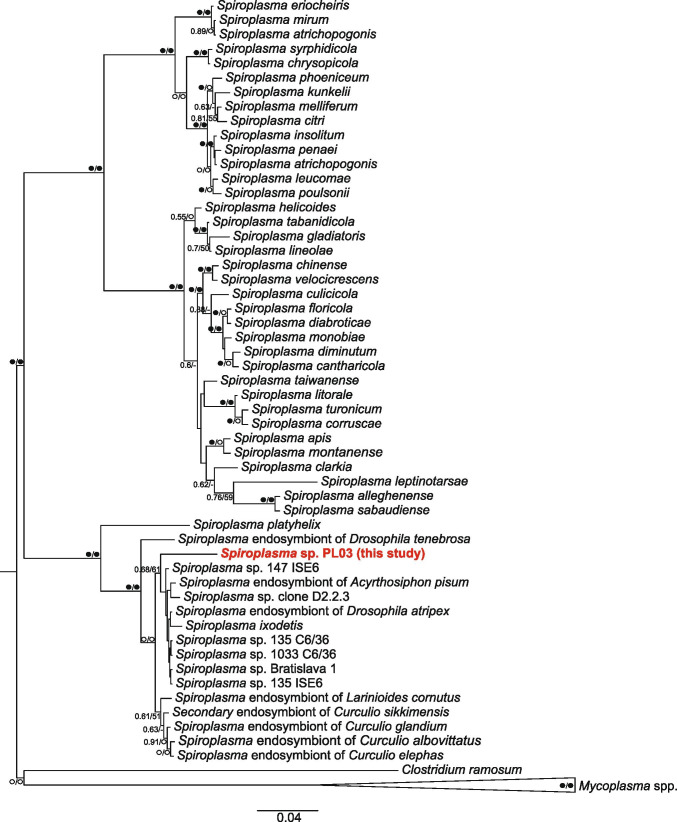
Phylogenetic tree of *Spiroplasma* 16S rRNA gene sequences inferred from BI and ML analyses. Values near branches show Bayesian posterior probabilities (PP) and bootstrap support values (BS) (PP/BS). Black circles: maximally supported; empty circles: supported > 0.95 PP and > 75% BS. Sequence found in this study is in red

Among the *Spiroplasmataceae*, only one ZOTU, *Spiroplasma* sp. PL03, was clustered. The sequence represented a *Spiroplasma* species that does not have a reference sequence published in databases. Phylogenetic analysis grouped this sequence with other endosymbiotic *Spiroplasma* spp. (Fig. 3). The prevalence of this species was 100% in infected and uninfected mosquitoes except for *C*. *richiardii* in which *Spiroplasma* was detected only in one infected individual (1.11%) (Fig. 1, Supplementary Table 3) and its presence in microsporidian-positive mosquitoes was 2.47 to 23.79% higher than in non-infected ones (Fig. 1, Supplementary Table 4). This result had statistical significance in *Ae. vexans*, *O. cantans* and *O. sticticus* (Supplementary Table 5). However, we did not find a significant correlation between increasing numbers of microsporidian and *Spiroplasma* sp. sequence reads (0.1 ≤ *r* ≤ 0.27, *p* = 0.216) (Supplementary Table 6).

Based on the 16S microbial profiling, the only representative ZOTU among *Leuconostocaceae* was assigned to the genus *Weissella*, while the almost-complete 16S rRNA gene (GenBank acc. nos. MW892051–MW892067) allowed species identification using BLASTn search, revealing the highest similarity to *Weissella viridescens* (100% identities). This bacterium was detected only in microsporidian-positive mosquitoes (Supplementary Fig. 4, Supplementary Table 4). There were no significant differences in *W.* cf. *viridescens* abundance among mosquito species (χ^2^ = 4.06, *p* = 0.4), but a strong positive correlation (0.45 ≤ *r* ≤ 0.81, *p* < 0.001) between microsporidian and *W.* cf. *viridescens* frequency was observed (Supplementary Table 6). Furthermore, an increased presence of *Spiroplasma* sp. PL03 was observed in *O. annulipes* individuals in which microsporidians co-occurred with *W.* cf. *viridescens* (*Z* =  − 2.51, *p* = 0.007) (Supplementary Table 7).

### Functional Analysis of Mosquito Microbiota During Microsporidian Infection

In total, based on the 16S amplicon dataset, PICRUSt2 generated 183 functional pathways using KEGG pathway metadata. The PICRUSt2 metagenome predictions had NSTI scores ranging from 0.001 to 0.182. The mean NSTI value was 0.049 for infected mosquitoes and 0.044 for non-infected ones (Supplementary Table 8).

**Fig. 4:**
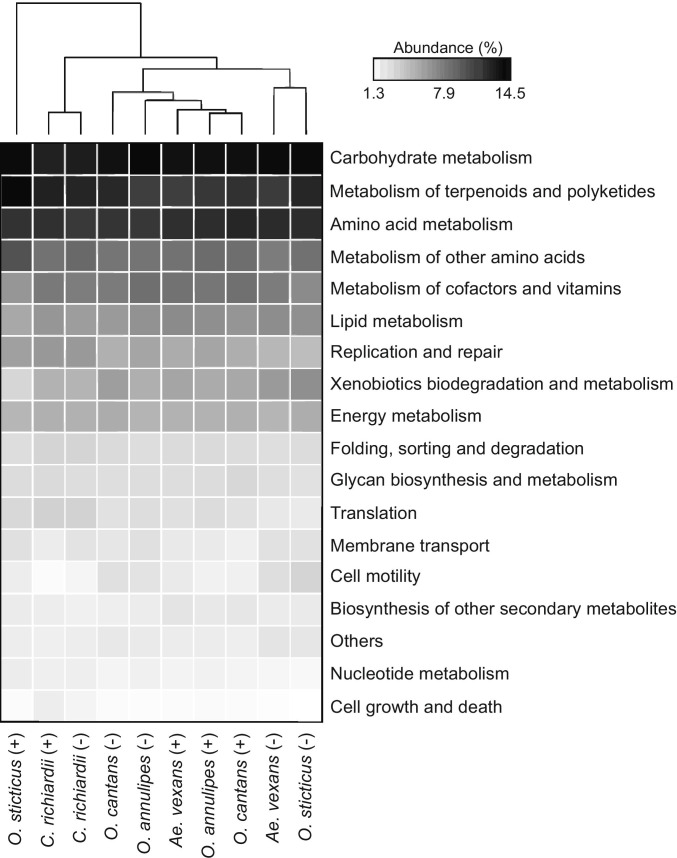
Heatmap of hierarchical clustering of relative abundance of the predicted pathways of the metagenome related to KEGG at level 2. Darker colour represents higher abundance in mosquito gut microbiome. Mosquito species are categorised as infected ( +) or non-infected (-)

Considering the total genes, almost 80% of them were related to the metabolic pathways and showed significant differences in abundance (Welch’s *t*-test, two-sided, *p* = 0.029) between microsporidian-positive and non-infected mosquitoes. For both infected and non-infected mosquitoes specifically, carbohydrate metabolism, metabolism of terpenoids and polyketides, and amino acid metabolism were the most abundant pathways (> 10%) at level 2 (Fig. 4, Supplementary Table 9).

**Fig. 5:**
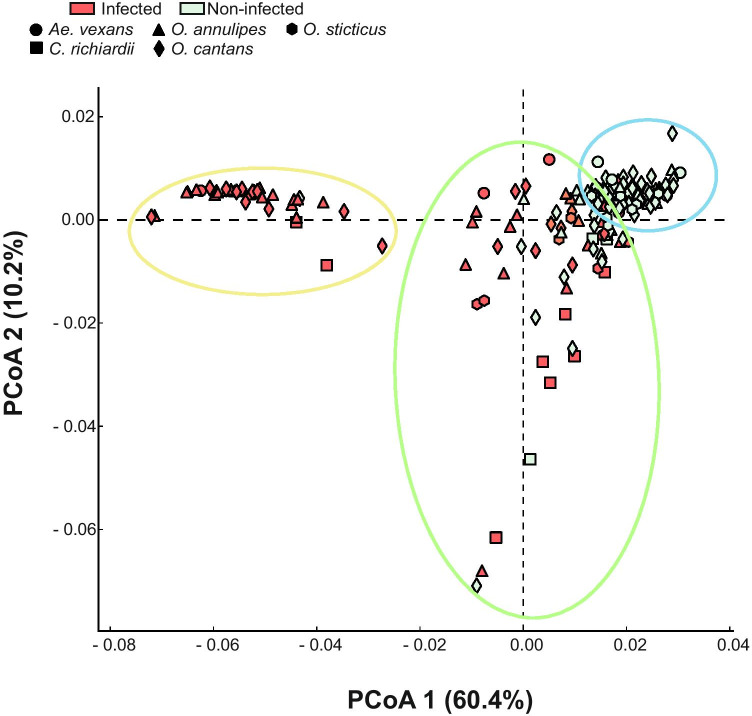
Pathways’ structure differentiation and inter-individual similarity based on the predicted pathways of the metagenome. PCoA was used to show patterns of separation. Point colour shows infection (red: infected; green: non-infected), and point shape identifies mosquito species (circle: *Aedes vexans*, square: *Coquillettidia richiardii*, triangle: *Ochlerotatus annulipes*, diamond: *O. cantans*, hexagon: *O. sticticus*). Ellipses shows groups of mosquitoes associated with the presence of microsporidians (yellow: mosquitoes associated with the presence of microsporidians, blue: mosquitoes not associated with the presence of microsporidians, green: both infected and non-infected mosquitoes)

Principal coordinate analysis based on microbiome predicted functions revealed three groups of mosquitoes that may be associated with the presence of microsporidians (Fig. 5). The first one included 35 microbiota found in microsporidian-infected mosquitoes, which represented all sampled mosquito species, and one microbiome detected in non-infected *O. cantans* individual. The second group consisted mostly of microbiota found in non-infected mosquitoes, also represented by all of the species. However, nine microbiota of infected individuals, including three each of *Ae. vexans, O. annulipes* and *O. cantans*, also belonged to this group. The last group contained both infected and non-infected mosquitoes.

**Fig. 6:**
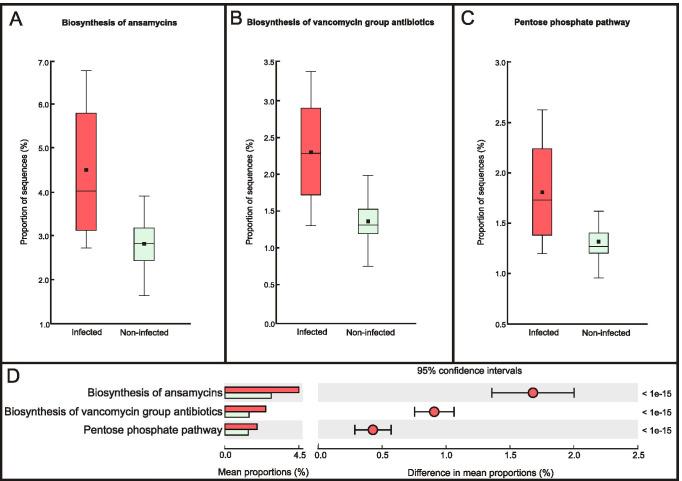
KEGG pathways at level 3 for the infected and non-infected mosquitoes by Microsporidia. Box plots of (**A**) biosynthesis of ansamycins, (**B**) biosynthesis of vancomycin group antibiotics, (**C**) pentose phosphate pathway and (**D**) extended error bar plot indicating differences in functional profiles

The microbiota of mosquitoes infected with microsporidians had a predicted metabolism more directed to the biosynthesis of ansamycins (*p* < 1e^−15^), the biosynthesis of vancomycin group antibiotics (*p* < 1e^−15^) and the pentose phosphate pathway (*p* < 1e^−15^), when compared to the microbiome of non-infected mosquitoes (Fig. 6). The proportion of these pathways relative to all pathways detected in infected mosquitoes was 8.0% (SD = 2.5%) and 5.5% (SD = 2.3%) in non-infected ones. The difference in mean proportion between these pathways ranged from 0.5% for the pentose phosphate pathway to 1.2% for the biosynthesis pathways of ansamycins.

## Discussion

### General Structure of Bacterial Communities

Our 16S rRNA microbial profiling results show that gut microbiota in field-collected mosquitoes sampled in Poland were dominated by Proteobacteria (about 60%) and Bacteroidetes and Firmicutes (each of about 20%) These results are broadly consistent with the findings of a large-scale study conducted in South Korea in which the gut bacteria of 305 insects, including mosquitoes, were examined [76]. Similarly, high contributions of Proteobacteria and Bacteroidetes were also found in the gut microbiota of insects sampled in Honolulu, Hawaii [77]. Moreover, we observed that the *Comamonadaceae*, *Enterobacteriaceae*, *Flavobacteriaceae* and *Pseudomonadaceae* are the largest contributors to the gut microbiota of both the infected and non-infected mosquitoes. The representatives of these families have also been identified during previous studies concerning mosquito microbiota [78–80].

The mosquito gut microbiome composition depends on factors that mainly affect the larval stages, such as environmental microbes, feeding behaviours, or biotic agents [81]. Mosquito larvae acquire bacteria during the feeding-filtering process or by vertical transmission [82–85]. It has been demonstrated that bacteria that have colonised the larvae are later found in adult individuals [83, 86, 87]. However, some evidence suggests that the habitat shift from aquatic to terrestrial may decrease the gut bacterial diversity [88, 89]. Moreover, diet amount and type, such as flower nectar, honeydew, fruits or blood, may also affect the adult mosquito microbiome structure. For example, in *Ae. aegypti*, a high diet abundance was positively correlated with *Enterobacteriaceae* and *Flavobacteriaceae* and negatively with *Sphingomonadaceae* [90]. The change in the microbiome composition as a result of the diet change was also observed in *An. gambiae*: bacteria belonging to the SAR11 clade were abundant in sugar-fed mosquitoes, while an increased presence of *Enterobacteriaceae*, *Yersiniaceae* and *Pseudomonadaceae* was noticed in mosquitoes after blood feeding [91]. However, most of the studies mentioned above focused on selected mosquito species and were conducted on laboratory-bred populations.

Reports on whether the mosquito microbiome composition can be species-dependent are sparse. Our ANCOM and UPGMA analyses suggest that adults in natural populations host microbial communities that vary among mosquito species. Similar observations have been provided for other Culicidae species, such as *Ae. japonicus*, *Ae. triseriatus*, *Culex coronator, Cx. nigripalpus* and *Cx. restuans* [81, 87]. It is well recognised that species specificity of gut microbiota has a biological basis, and some mechanisms underlying reciprocal host–bacteria selection have already been proposed (for review see [92]); however, future studies are needed because these reports are scarce and mainly involve vertebrates.

Although PCoA considering all mosquito microbiota as one pooled sample suggested that microsporidians do not affect the gut bacterial compositions, the analyses carried out for each species separately grouped mosquito microbiota according to the infection. An exception was *O. cantans*, for which we observed a partial overlap between the microbial compositions of infected and microsporidian-free individuals. Further studies, including quantitative analyses, should be performed to determine whether this result is associated with the infecting microsporidian species or the level of infestation.

### Gut Microbiome Members Associated with Microsporidian Infection

Our data show that infection by microsporidians is associated with a change in the gut microbial composition of the host. To our knowledge, no prior studies have examined the impact of microsporidian infections on the mosquito microbiota, while reports about correlations between microsporidians and microbiota of other invertebrate hosts are sparse and mainly concern honeybees. For instance, it has been noted that the abundance of *Alphaproteobacteria* decreased in *Ap. mellifera* honey bees infected with *N. ceranae* and that this effect was stronger when the infected bees were chemically exposed to insecticides or fungicides; additionally, the *Gammaproteobacteria* abundance increased in the *Nosema*-infected bees, but significantly only when hosts were co-exposed to pesticides [93]. Our results are partially consistent with these findings. We also noted the decreased abundance of *Alphaproteobacteria* in microsporidian-infected *Co. richiardii*, *O. cantans* and *O. sticticus*. In addition, we found an increased contribution of *Gammaproteobacteria* in infected *O. annulipes* and *O. sticticus*.

Our results suggest that microsporidian infections are correlated with specific gut microbiome members. The occurrence of *W.* cf. *viridescens* was unequivocally correlated with infection. The genus *Weissella* belongs to lactic acid bacteria [94–96]. Although they are somewhat ubiquitous in the environment, such as soil, lakes and sediments of a coastal marsh [94–96], *Weissella* spp. are also found as components of insect gut microbiota, for example, of Hymenoptera and Orthoptera [16, 97]. Moreover, it has been shown that microsporidians may reduce the pH in the gut, inhibiting the growth of most bacteria, except for acid-tolerant species [16]. Our results concur with these observations because *Weissella* spp. can survive in an acidic environment [16, 94–96].

The occurrence of *Spiroplasma* sp. PL03 and *W.* cf. *viridescens* in microsporidia-positive mosquitoes suggests associations between these bacteria and parasitic infection. *Spiroplasma* is broadly distributed among invertebrate hosts, such as insects and spiders [98–101]. Additionally, some endosymbiotic *Spiroplasma* spp. in insects can confer resistance to a range of parasites, including fungi, nematodes and parasitoids [98–100]. Although most members of the genus are endosymbionts, some species can be strongly virulent in male hosts [100, 101]. The seven *Spiroplasma* species isolated, to date, from mosquitoes are not virulent to mosquitoes (Supplementary Table 10). Therefore, greater participation of *Spiroplasma* sp. PL03 in microsporidian-positive mosquitoes than in non-infected ones suggests an association of these bacteria with microsporidian infection. Our phylogenetic analyses confirmed that the identified *Spiroplasma* sp. PL03 is an endosymbiotic species; however, many questions remain unresolved, such as transmission mode, tissue tropism and fitness effects [98].

### Functional Analysis of Mosquito Microbiota During Microsporidian Infection

The prediction of metagenome functional content from the 16S rRNA gene sequencing suggests that during microsporidian infection, the mosquito microbiome is relatively more abundant in bacterial species capable of metabolising terpenoids and polyketides and synthesising ansamycin and vancomycin group antibiotics. The oxidative phosphorylation and pentose phosphate pathways are also comparatively more abundant in the microbiota of infected hosts.

The increased ability to synthesise antibiotics appears to be a natural mechanism to protect the host already weakened by parasitic infection. Ansamycins form a class of bacterial macrocyclic polyketides that exhibit broad inhibitory activities, including antibacterial, antifungal, antiviral and immunosuppressant [102, 103]. For example, rifamycin, a naphthalenic ansamycin, inhibits RNA transcription in many bacterial species [104, 105]. Vancomycin is a branched tricyclic glycopeptide antibiotic that inhibits cell wall synthesis and is active against most Gram-positive bacteria, including anaerobic clostridia [106, 107]. *Weissella* cf. *viridescens* belongs to the family *Leuconostocaceae*, whose members are intrinsically vancomycin-resistant [94, 95, 108]. Therefore, the vancomycin synthesis could explain the presence of *Weissella* only in microsporidian-infected mosquitoes.

Microsporidians have lost most of the genes needed for making primary metabolites, such as nucleotides and amino acids, and have a limited capacity to generate adenosine triphosphate (ATP) [16, 109, 110]. The loss of key pathways for energy generation has resulted in microsporidians having to obtain those substrates from host cells [111]. Moreover, He et al. [109] showed that after the induction of spore germination, the majority of the microsporidian genes involved in the pentose phosphate pathway are downregulated. Thus, they suggested that sporoplasm might inhibit its carbon metabolic activity and obtain the substances required for proliferation from host cells. Our observation that the pentose phosphate pathways are relatively more abundant in the microbiota of microsporidian-infected mosquitoes supports the hypothesis that microsporidians might manipulate biological processes in their environment to promote nucleotide synthesis and maximise the potential for ATP and nucleotide import [13, 109]. However, we acknowledge that our results cannot resolve whether the predicted observed alteration in gut microbial functionalities is a microsporidian-mediated response or caused by the host immune system or due to a change in the mycobiome. Therefore, future studies should be carried out, including mycobiome identification and host transcriptome analysis.

## Conclusion

In this paper, we suggest that microsporidian infection shapes the microbial community structure and microbial activity in infected mosquitoes, especially the biosynthesis of antibiotics and the pentose phosphate pathway. This result confirms previous findings that parasites modulate the gut microbiome in insects and that this is an important agent during modulated host- and parasite-associated microbiome interactions. Endosymbiotic *Spiroplasma* sp. PL03 and *W.* cf. *viridescens*, two bacterial species found in this study, represent bacteria whose participation in the mosquito gut is highly dependent on microsporidian infection. Our results imply that the gut microbiome may also affect pathogens vectored by mosquitoes. For example, the previously observed resistance of microsporidian-infected mosquitoes to *Plasmodium* transmission [10] might be related to changes in the structure and metabolism of the mosquito microbiome. Considering that the oocysts must nest in the gut, settled beneath the epithelium, and attached to the basal lamina, the alterations in the intestinal microbiome caused by microsporidian infection cannot be excluded as one of the mechanisms preventing the parasite’s development. However, more extensive research, including the determination of metabolic activity based on quantitative methods and experimental infection under controlled conditions, should be carried out to test this hypothesis.

## Supplementary Information

Below is the link to the electronic supplementary material.

Supplementary file1 (PDF 837 KB)
