# Supplementary material for: Microsporidian Infection in Mosquitoes (Culicidae) Is Associated with Gut Microbiome Composition and Predicted Gut Microbiome Functional Content
Source: Microb Ecol. 2021 Dec 23;85(1):247–63. doi: 10.1007/s00248-021-01944-z (PMC9849180; doi:10.1007/s00248-021-01944-z)
Supplement: Supplementary file 1 — Supplementary file1 (PDF 837 KB) [file 248_2021_1944_MOESM1_ESM.pdf]

Supplementary Information for:

Microsporidian infection changes mosquito microbiota and affects specific gut microbiome members

Artur Trzebny[1*], Anna Slodkowicz-Kowalska[2], Johanna Björkroth[3], Miroslawa Dabert[1]

[1] Molecular Biology Techniques Laboratory, Faculty of Biology, Adam Mickiewicz University, Poznan, Poland

[2] Department of Biology and Medical Parasitology, Poznan University of Medical Sciences, Poznan, Poland

[3] Department of Food Hygiene and Environmental Health, Faculty of Veterinary Medicine, University of Helsinki, Helsinki, Finland

* **Corresponding author:**

Artur Trzebny: Molecular Biology Techniques Laboratory, Faculty of Biology, Adam Mickiewicz University, Poznan, Poland, E-mail: arturtrzebny@amu.edu.pl

Table of Contents:

Supplementary Information	Page
Supplementary Figure 1	Page 3
Supplementary Figure 2	Page 4
Supplementary Figure 3	Page 5
Supplementary Figure 4	Page 6
Supplementary Table 1	Page 7
Supplementary Table 2	Page 10
Supplementary Table 3	Page 10
Supplementary Table 4	Page 11
Supplementary Table 5	Page 13
Supplementary Table 6	Page 14
Supplementary Table 7	Page 14
Supplementary Table 8	Page 15
Supplementary Table 9	Page 16
Supplementary Table 10	Page 18
Supplementary Bibliography	Page 19

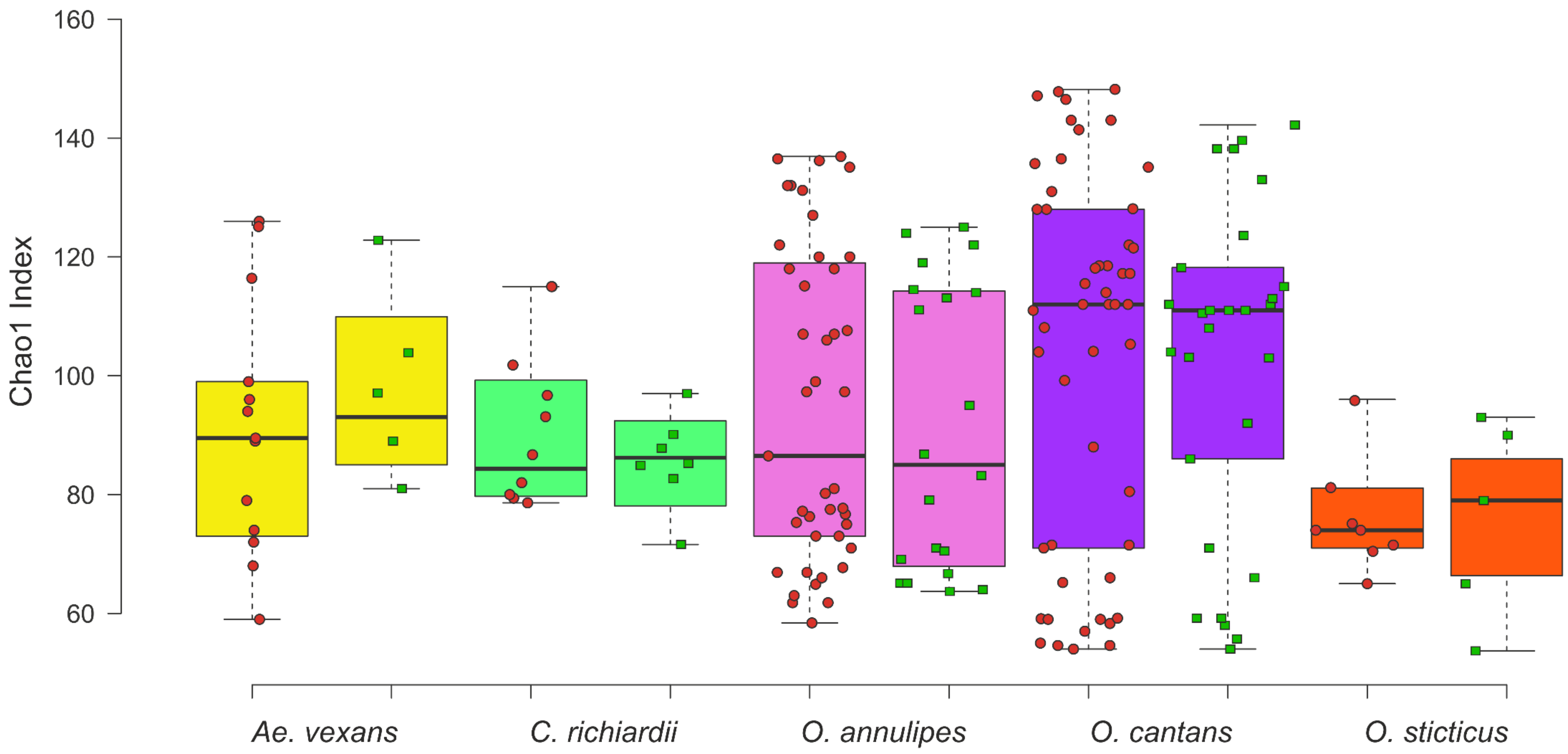

Supplementary Figure 1. Boxplot representation of Chao1 showing the distribution of bacteria between mosquito species categorised as infected (red circles) and non-infected (green squares).

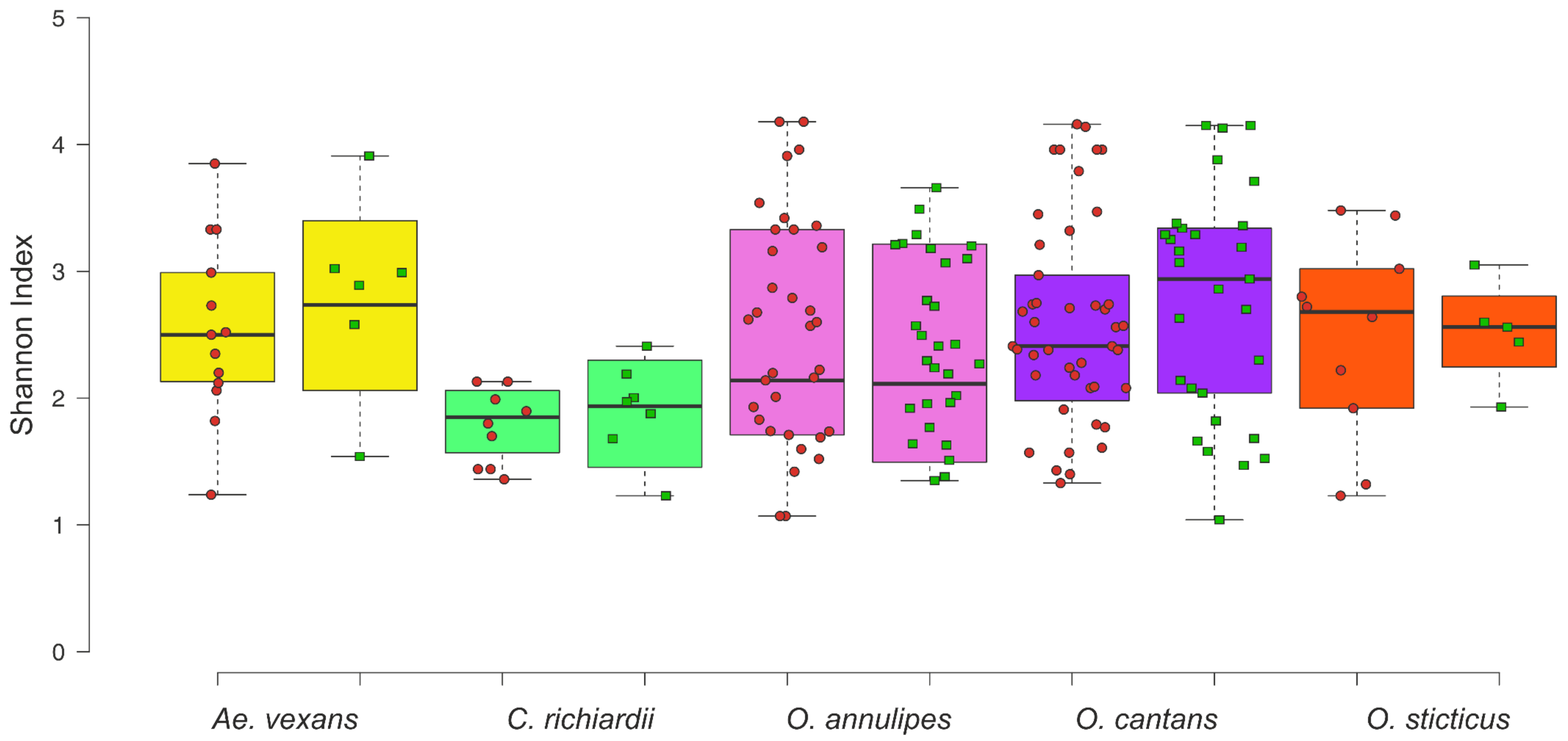

Supplementary Figure 2. Boxplot representation of Shannon diversity shows the distribution of bacteria between mosquito species categorised as infected (red circles) and non-infected (green squares).

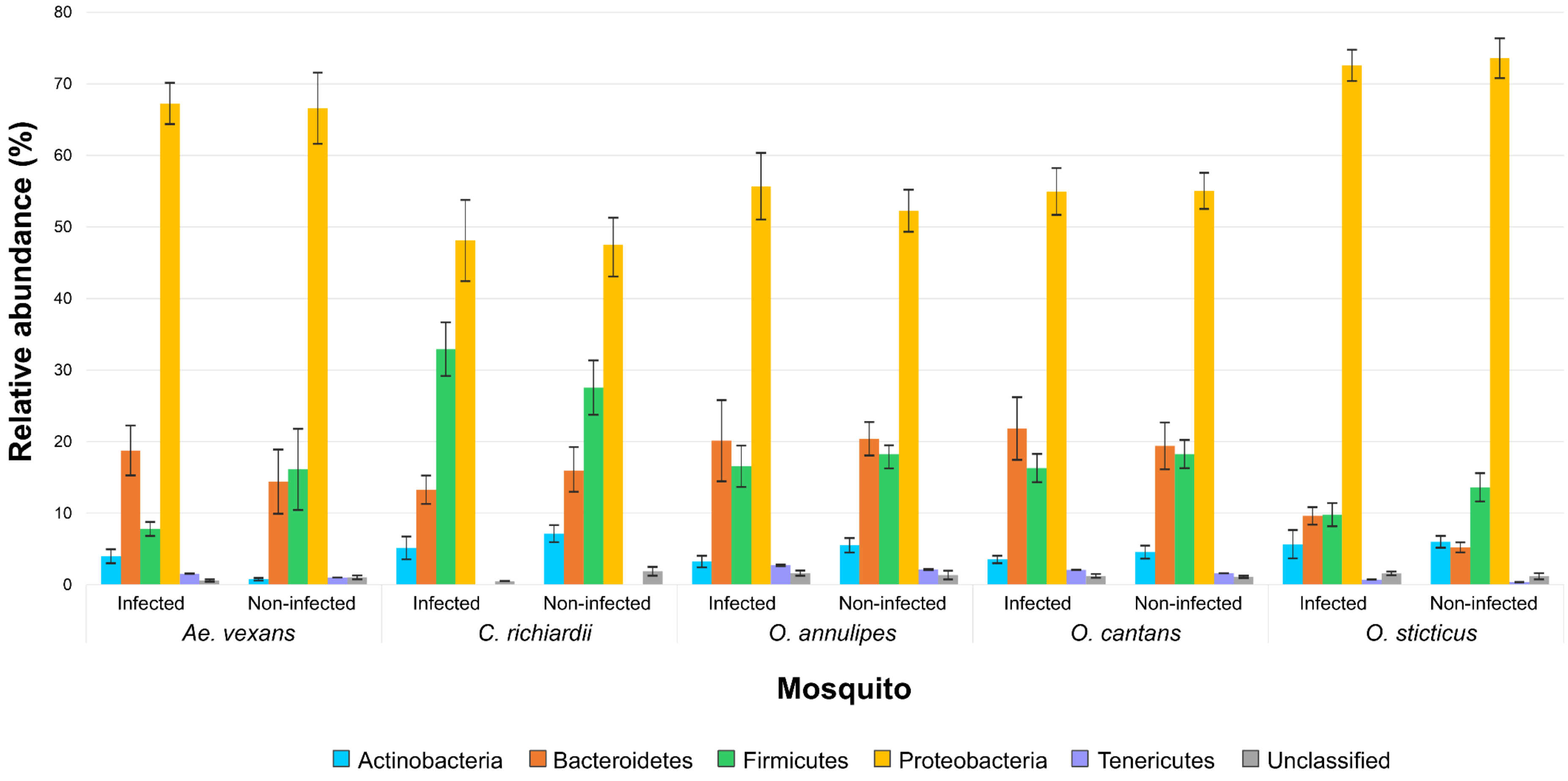

Supplementary Figure 3. Mean relative abundances of bacterial phyla among infected and non-infected mosquitoes.

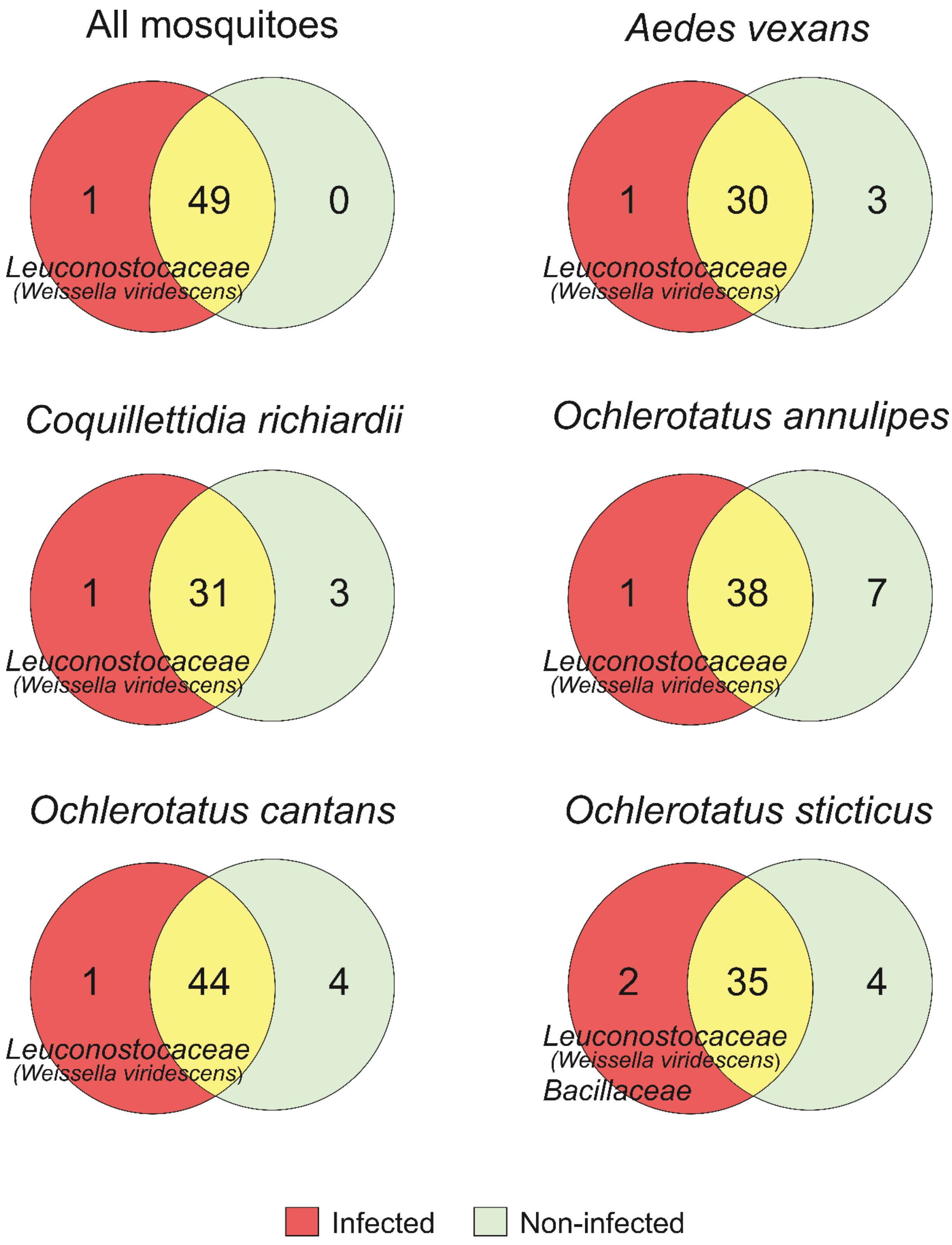

Supplementary Figure 4. Venn diagrams showing the numbers of unique and shared bacterial families across infected and non-infected mosquitoes.

Supplementary Table 1. Bacterial strain and GenBank accession numbers for DNA sequences used in the *Spiroplasma* spp. phylogenetic analysis.

Group	Species	Strain	GenBank no.
Spiroplasma	*Spiroplasma alleghenense*	PLHS-1; ATCC 51752	NR_025697.1
Spiroplasma	*Spiroplasma apis*	B31	NR_104858.1
Spiroplasma	*Spiroplasma atrichopogonis*	GNAT3597	NR_104720.1
Spiroplasma	*Spiroplasma cantharicola*	CC-1	NR_125516.1
Spiroplasma	*Spiroplasma chinense*	CCH; ATCC 43960	NR_025698.1
Spiroplasma	*Spiroplasma chrysopicola*	ATCC 43209; DF-1	AY189127.1
Spiroplasma	*Spiroplasma citri*	R8A2HP	NR_036849
Spiroplasma	*Spiroplasma clarkia*	DSM19994T	FR733690.1
Spiroplasma	*Spiroplasma corruscae*	EC-1; ATCC 43212	NR_025700.1
Spiroplasma	*Spiroplasma culicicola*	AES-1; ATCC 35112	NR_025701.1
Spiroplasma	*Spiroplasma diabroticae*	DU-1	NR_104751.1
Spiroplasma	*Spiroplasma diminutum*	ATCC 49235; CUAS-1	AY189130.1
Spiroplasma	*Spiroplasma* endosymbiont of *Acyrthosiphon pisum*	185	JX943566.1
Spiroplasma	*Spiroplasma* endosymbiont of *Curculio albovittatus*	----	AB604655.1
Spiroplasma	*Spiroplasma* endosymbiont of *Curculio elephas*	J73E	JN100091.1
Spiroplasma	*Spiroplasma* endosymbiont of *Curculio glandium*	M1_H2	JQ692307.1
Spiroplasma	*Spiroplasma* endosymbiont of *Curculio sikkimensis*	----	AB545038.1

Group	Species	Strain	GenBank no.
Spiroplasma	*Spiroplasma* endosymbiont of *Drosophila atripex*	----	FJ657246.1
Spiroplasma	*Spiroplasma* endosymbiont of *Drosophila tenebrosa*	----	FJ657241.1
Spiroplasma	*Spiroplasma* endosymbiont of *Larinioides cornutus*	----	KX559380.1
Spiroplasma	*Spiroplasma eriocheiris*	CRAB	NR_125505.1
Spiroplasma	*Spiroplasma floricola*	ATCC 29989; 23-6	NR_025703.1
Spiroplasma	*Spiroplasma gladiatoris*	TG-1	NR_044670.2
Spiroplasma	*Spiroplasma helicoides*	TABS-2; ATCC 51746	NR_025704.1
Spiroplasma	*Spiroplasma insolitum*	M55; ATCC 33502	NR_025705.1
Spiroplasma	*Spiroplasma ixodetis*	Y32	NR_104852.1
Spiroplasma	*Spiroplasma kunkelii*	E275	NR_104847.1
Spiroplasma	*Spiroplasma leptinotarsae*	E275	NR_104847.1
Spiroplasma	*Spiroplasma leucomae*	SMA	NR_104721.1
Spiroplasma	*Spiroplasma lineolae*	TALS-2	NR_125515.1
Spiroplasma	*Spiroplasma litorale*	TN-1; ATCC 34211	NR_025708.1
Spiroplasma	*Spiroplasma melliferum*	BC-3; ATCC 33219	NR_025756.1
Spiroplasma	*Spiroplasma mirum*	SMCA	NR_104955.1
Spiroplasma	*Spiroplasma monobiae*	MQ-1	NR_104854.1
Spiroplasma	*Spiroplasma montanense*	HYOS-1; ATCC 51745	NR_025709.1
Spiroplasma	*Spiroplasma penaei*	SHRIMP	NR_043177.1
Spiroplasma	*Spiroplasma phoeniceum*	P40	NR_043178.1

Group	Species	Strain	GenBank no.
Spiroplasma	*Spiroplasma platyhelix*	PALS-1	NR_104857.1
Spiroplasma	*Spiroplasma poulsonii*	DW-1	NR_044672.1
Spiroplasma	*Spiroplasma sabaudiense*	Ar-1343; ATCC 43303	NR_025710.1
Spiroplasma	*Spiroplasma* sp. 1033 C6/36	----	LC388770.1
Spiroplasma	*Spiroplasma* sp. 135 C6/36	----	LC388760.1
Spiroplasma	*Spiroplasma* sp. 135 ISE6	----	LC388759.1
Spiroplasma	*Spiroplasma* sp. 147 ISE6	----	LC388762.1
Spiroplasma	*Spiroplasma* sp. Bratislava 1	Bratislava 1	KP967685.1
Spiroplasma	*Spiroplasma* sp. clone D2.2.3	----	AY837745.1
Spiroplasma	*Spiroplasma syrphidicola*	EA-1	AY549209.1
Spiroplasma	*Spiroplasma tabanidicola*	TAUS-1	NR_104753.1
Spiroplasma	*Spiroplasma taiwanense*	CT-1	NR_121701.1
Spiroplasma	*Spiroplasma turonicum*	Tab-4c; ATCC 700271	NR_025712.1
Spiroplasma	*Spiroplasma velocicrescens*	MQ-4; ATCC 35262	NR_025713.1
Outgrup	*Clostridium ramosum*	113-T	NR_029247.1
Outgrup	*Mycoplasma californicum*	ST-6; ATCC 33461	NR_029166.1
Outgrup	*Mycoplasma crocodyli*	MP145	NR_074301.1
Outgrup	*Mycoplasma hominis*	PG21; ATCC 23114	NR_041881.1
Outgrup	*Mycoplasma lagogenitalium*	12MS; ATCC 700289	NR_025185.1
Outgrup	*Mycoplasma synoviae*	WVU 1853	NR_044811.1

Supplementary Table 2. Mean relative abundances of phyla-level bacteria among infected and non-infected mosquitoes.

Phylum	*Ae. vexans*		*C. richiardii*		*O. annulipes*		*O. cantans*		*O. sticticus*	
	**Infected (SD)**	**Non-infected (SD)**	**Infected (SD)**	**Non-infected (SD)**	**Infected (SD)**	**Non-infected (SD)**	**Infected (SD)**	**Non-infected (SD)**	**Infected (SD)**	**Non-infected (SD)**
Actinobacteria	3.99 (2.16)	0.78 (0.33)	5.16 (3.92)	7.14 (2.92)	3.24 (1.74)	5.52 (2.6)	3.54 (1.42)	4.56 (1.77)	5.67 (4.41)	6 (2.23)
Bacteroidetes	18.78 (7.21)	14.42 (9.7)	13.29 (4.63)	15.92 (7.49)	20.13 (11.03)	20.42 (8.79)	21.85 (12.52)	19.41 (11.26)	9.62 (3.5)	5.22 (2.13)
Firmicutes	7.81 (2.21)	16.14 (11.86)	32.93 (7.7)	27.56 (9.26)	16.57 (5.49)	18.28 (6.49)	16.31 (5.54)	18.27 (5.67)	9.81 (6.41)	13.64 (6.46)
Proteobacteria	67.27 (6.12)	66.6 (10.13)	48.12 (12.38)	47.49 (9.63)	55.7 (10.43)	52.28 (9.65)	54.96 (11.06)	55.05 (9.55)	72.59 (9)	73.59 (7.85)
Tenericutes	1.54 (0.01)	1.02 (<0.01)	0.00	0.00	2.72 (0.03)	2.13 (0.02)	2.1 (0.02)	1.61 (0.01)	0.72 (0.01)	0.35 (<0.01)
Unclassified	0.61 (0.29)	1.04 (0.41)	0.5 (<0.01)	1.89 (0.85)	1.64 (0.45)	1.37 (0.81)	1.24 (0.74)	1.1 (0.45)	1.59 (0.74)	1.2 (0.89)

Supplementary Table 3. Prevalence of *Spiroplasma* sp. PL03 among infected and non-infected mosquitoes.

Species	*Ae. vexans*		*C. richiardii*		*O. annulipes*		*O. cantans*		*O. sticticus*	
	**Infected (%)**	**Non-infected (%)**	**Infected (%)**	**Non-infected (%)**	**Infected (%)**	**Non-infected (%)**	**Infected (%)**	**Non-infected (%)**	**Infected (%)**	**Non-infected (%)**
Spiroplasma sp. PL03	13/13 (100)	6/6 (100)	1/9 (1.11)	0/7 (0)	34/34 (100)	29/29 (100)	44/44 (100)	33/33 (100)	8/8 (100)	5/5 (100)

Supplementary Table 4. Mean relative abundances of family-level bacteria among infected and non-infected mosquitoes.

Phylum	Family	*Ae. vexans*		*C. richiardii*		*O. annulipes*		*O. cantans*		*O. sticticus*	
		Infected (SD)	Non-infected (SD)	Infected (SD)	Non-infected (SD)	Infected (SD)	Non-infected (SD)	Infected (SD)	Non-infected (SD)	Infected (SD)	Non-infected (SD)
Actinobacteria	*Micrococcaceae*	3.27 (2.06)	0.39 (0.24)	4.31 (3.7)	4.44 (2.76)	1.84 (1.56)	3.4 (2.15)	1.34 (0.92)	2.33 (1.49)	2.61 (1.67)	1.37 (0.01)
Actinobacteria	*Actinomycetales*	0.25 (0.14)	0.09 (0.13)	0.36 (0.3)	0.76 (0.64)	0.22 (0.15)	0.55 (0.41)	0.28 (0.19)	0.49 (0.29)	0.62 (0.15)	0.09 (0.21)
Actinobacteria	*Corynebacteriaceae*	0.23 (0.16)	0.09 (0.07)	0.35 (0.2)	1.07 (0.82)	0.7 (0.48)	1.13 (0.88)	0.81 (0.49)	0.68 (0.41)	1.4 (1.84)	0.55 (0.25)
Actinobacteria	*Patulibacteraceae*	0.2 (0.1)	0.15 (0.09)	0 (0)	0.01 (0.01)	0.08 (0.06)	0.07 (0.05)	0.19 (0.13)	0.06 (0.04)	0 (0)	0.1 (0.16)
Actinobacteria	*Mycobacteriaceae*	0.02 (0.02)	0.02 (0.01)	0.05 (0.03)	0.37 (0.22)	0.1 (0.08)	0.2 (0.09)	0.5 (0.36)	0.49 (0.28)	0.22 (0.13)	0.24 (0.77)
Actinobacteria	*Conexibacteraceae*	0.02 (0.01)	0.02 (0.02)	0 (0)	0 (0)	0 (0)	0 (0)	0.03 (0.02)	0.07 (0.05)	0 (0)	0 (0)
Actinobacteria	*Actinomycetaceae*	0 (0)	0.02 (0.01)	0.02 (0.02)	0.12 (0.07)	0.01 (0.01)	0.07 (0.06)	0.03 (0.03)	0.01 (0.01)	0.25 (0.24)	0.44 (0.19)
Actinobacteria	*Bifidobacteriaceae*	0 (0)	0 (0)	0 (0)	0 (0)	0.01 (0.01)	0.04 (0.02)	0.01 (0.01)	0.04 (0.03)	0.01 (0.86)	0.01 (0.05)
Actinobacteria	*Brevibacteriaceae*	0 (0)	0 (0)	0 (0)	0 (0)	0.09 (0.06)	0.02 (0.02)	0 (0)	0.05 (0.03)	0.26 (<0.01)	2.83 (0.01)
Actinobacteria	*Dermabacteraceae*	0 (0)	0 (0)	0 (0)	0 (0)	0.01 (0.01)	0 (0)	0 (0)	0.04 (0.03)	0 (0)	0.01 (0.22)
Actinobacteria	*Nocardiaceae*	0 (0)	0 (0)	0.06 (0.03)	0.34 (0.21)	0.14 (0.12)	0.02 (0.01)	0.34 (0.24)	0.28 (0.19)	0.3 (0.31)	0.2 (0.14)
Actinobacteria	*Pseudonocardiaceae*	0 (0)	0 (0)	0.01 (0.01)	0.03 (0.03)	0.04 (0.04)	0.02 (0.01)	0.01 (0.01)	0.02 (0.01)	0 (0)	0.16 (0.04)
Bacteroidetes	*Flavobacteriaceae*	18.73 (7.22)	14.08 (9.78)	13.25 (4.63)	14.78 (7.86)	18.22 (10.99)	19.14 (8.87)	21.04 (12.68)	18.92 (11.25)	8.58 (3.8)	3.94 (1.49)
Bacteroidetes	*Porphyromonadaceae*	0.03 (0.02)	0.3 (0.22)	0.02 (0.01)	1.07 (0.7)	1.67 (1.25)	1.26 (0.89)	0.72 (0.46)	0.28 (0.15)	1.04 (0.51)	1.25 (2.23)
Bacteroidetes	*Chitinophagaceae*	0.02 (0.01)	0.04 (0.03)	0.02 (0.01)	0.07 (0.06)	0.24 (0.14)	0.02 (0.01)	0.09 (0.06)	0.21 (0.18)	0 (0)	0.03 (0.14)
Bacteroidetes	*Cytophagaceae*	0 (0)	0 (0)	<0.01 (<0.01)	0 (0.01)	0 (0)	0 (0)	0 (0)	0 (0)	0 (0)	<0.01 (<0.01)
Firmicutes	*Staphylococcaceae*	3.12 (1.38)	12.68 (11.34)	5.25 (2.24)	0.89 (0.77)	2.02 (1.49)	7.15 (5.81)	4.82 (3.03)	3.35 (2.12)	2.89 (4.04)	2.69 (0.02)
Firmicutes	*Leuconostocaceae*	2.07 (0)	0 (0)	2.59 (0.01)	0 (0)	3.96 (<0.01)	0 (0)	1.97 (<0.01)	0.00	1.17 (<0.01)	0 (0)
Firmicutes	*Ruminococcaceae*	2.04 (1.35)	2.4 (0.59)	2.5 (1.58)	1.99 (1.24)	4.72 (3.02)	7.02 (4.59)	5.54 (3.35)	10.51 (6.22)	3.67 (1.96)	7.26 (4.2)
Firmicutes	*Streptococcaceae*	0.43 (0.36)	0.94 (0.4)	9.81 (5.8)	8.01 (6.38)	1.37 (1.05)	1.96 (1.15)	1.79 (1.22)	2.12 (1.43)	1.29 (1.83)	2.86 (5.45)
Firmicutes	*Lactobacillaceae*	0.15 (0.1)	0.06 (0.05)	0 (0)	0 (0)	3.94 (3.28)	1.13 (0.67)	1.14 (0.81)	1.66 (0.85)	0 (0)	<0.01 (0.04)
Firmicutes	*Bacillaceae*	0 (0)	0 (0)	0.09 (0.06)	0.11 (0.09)	0.05 (0.03)	0.02 (0.01)	0.04 (0.03)	0.15 (0.1)	0.26 (0.18)	0 (0)
Firmicutes	*Carnobacteriaceae*	0 (0)	0 (0)	0 (0)	0.01 (0.01)	0.2 (0.16)	0.02 (0.01)	0.04 (0.03)	0.09 (0.05)	0 (0)	0 (0)
Firmicutes	*Clostridiales*	0 (0)	0.06 (0.04)	0 (0)	0 (0)	0 (0)	0.28 (0.18)	0.33 (0.21)	0.15 (0.1)	0.12 (0.08)	0.76 (0.17)

Phylum	Family	*Ae. vexans*		*C. richiardii*		*O. annulipes*		*O. cantans*		*O. sticticus*	
		Infected (SD)	Non-infected (SD)	Infected (SD)	Non-infected (SD)	Infected (SD)	Non-infected (SD)	Infected (SD)	Non-infected (SD)	Infected (SD)	Non-infected (SD)
Firmicutes	*Enterococcaceae*	0 (0)	0 (0)	12.69 (4.65)	16.55 (8.99)	0.31 (0.22)	0.7 (0.4)	0.64 (0.53)	0.24 (0.17)	0.41 (0.25)	0.07 (0.7)
Proteobacteria	*Comamonadaceae*	9.16 (4.65)	8.34 (4.16)	7.41 (4.9)	6.24 (3.62)	9.43 (8.41)	12.02 (7.61)	8.74 (5.52)	6.51 (4)	8.99 (4.72)	8.62 (0.22)
Proteobacteria	*Acetobacteraceae*	8.05 (3.58)	6.25 (4.26)	2.33 (1.46)	1.43 (0.97)	7.12 (3.64)	3.25 (2.02)	6.22 (3.77)	5.41 (3.03)	6.35 (4.47)	6.69 (0.95)
Proteobacteria	*Methylobacteriaceae*	5.69 (3.21)	6.14 (3.44)	0.09 (0.16)	0.29 (0.16)	3.17 (2.47)	2.38 (1.52)	2.01 (1.32)	5.91 (3.89)	0.06 (0.05)	0.18 (0.18)
Proteobacteria	*Bradyrhizobiaceae*	3.02 (3.09)	2.87 (1.42)	2.06 (1.16)	0.72 (0.45)	1.34 (1.14)	0.8 (0.44)	2.84 (1.93)	0.93 (0.6)	0.3 (0.15)	0.37 (4.17)
Proteobacteria	*Rhizobiaceae*	2.05 (0.99)	2.16 (1.22)	0.17 (0.18)	0.94 (0.95)	1.3 (0.89)	0.94 (0.51)	2.55 (1.66)	1.17 (0.78)	0.24 (0.38)	0.15 (0.13)
Proteobacteria	*Enterobacteriaceae*	19.56 (7.16)	28.32 (9.15)	19.45 (11.84)	21.26 (9.78)	19.51 (11.56)	17.56 (8.68)	15.85 (8.33)	19.94 (9.97)	38.55 (11.98)	34.69 (6.65)
Proteobacteria	*Proteobacteria*	15.97 (9.02)	9.54 (4.55)	0.45 (0.38)	0.9 (0.42)	9.06 (7.05)	7.08 (4.68)	4.98 (3.38)	2.37 (1.81)	4.62 (4.2)	6.73 (2.42)
Proteobacteria	*Moraxellaceae*	1.27 (0.95)	1.33 (0.45)	0 (0)	0.89 (0.53)	0.36 (0.29)	0.63 (0.38)	0.24 (0.13)	1.2 (0.74)	0.66 (0.94)	1.76 (0.22)
Proteobacteria	*Xanthomonadaceae*	1.04 (0.44)	0.68 (0.62)	0.35 (0.2)	0.86 (0.93)	0.75 (0.63)	0.9 (0.67)	0.93 (0.53)	0.75 (0.51)	1.08 (0.8)	0.92 (0.6)
Proteobacteria	*Pseudomonadaceae*	0.5 (0.36)	0.34 (0.25)	6.21 (3.02)	3.92 (1.51)	1.96 (1.62)	1.73 (1.19)	3.73 (2.65)	1.8 (1.05)	10.19 (4.89)	8.43 (6.29)
Proteobacteria	*Sphingomonadaceae*	0.35 (0.19)	0.08 (0.25)	0.13 (0.11)	2.01 (1.41)	0.85 (0.55)	2 (1.27)	0.26 (0.18)	1.17 (0.85)	0.53 (0.27)	0.32 (0.24)
Proteobacteria	*Caulobacteraceae*	0.25 (0.11)	0.02 (0.21)	4.12 (3.75)	3.87 (2.42)	0.6 (0.44)	1.07 (0.71)	1.3 (0.89)	1.79 (0.96)	0.1 (0.14)	2.26 (0.11)
Proteobacteria	*Halomonadaceae*	0.18 (0.11)	0.06 (0.07)	4.68 (2.89)	3.94 (1.38)	0 (0)	0.81 (0.53)	0.04 (0.03)	0.09 (0.06)	0.26 (0.32)	0.39 (0.86)
Proteobacteria	*Rhizobiales*	0.12 (0.05)	0.15 (0.12)	0 (0)	0 (0)	0.04 (0.04)	0.02 (0.01)	0.04 (0.03)	0.09 (0.06)	0 (0)	0 (0)
Proteobacteria	*Hydrogenophilaceae*	0.02 (0.01)	0.11 (0.07)	0.02 (0.02)	0.02 (0.04)	0 (0)	0.1 (0.08)	0 (0)	0.02 (0.01)	0.02 (0.03)	0.39 (0.23)
Proteobacteria	*Oxalobacteraceae*	0.02 (0.01)	0.02 (0.01)	0.48 (0.31)	0.01 (0.01)	0.04 (0.03)	0.03 (0.02)	0.05 (0.03)	0.04 (0.02)	0.04 (0.01)	0.02 (1.05)
Proteobacteria	*Phyllobacteriaceae*	0.02 (0.01)	0.15 (0.05)	0 (0)	0 (0)	0 (0)	0.25 (0.14)	4.48 (3.07)	3.84 (2.67)	0 (0)	0 (0)
Proteobacteria	*Aurantimonadaceae*	0 (0)	0 (0)	0 (0)	0 (0)	0.03 (0.03)	0.03 (0.02)	0 (0)	0.11 (0.07)	0.06 (0.05)	0.17 (2.54)
Proteobacteria	*Coxiellaceae*	0 (0)	0 (0)	0.17 (0.1)	0.19 (0.08)	0.02 (0.02)	0.04 (0.03)	0.34 (0.23)	0.44 (0.25)	0 (0)	0 (0)
Proteobacteria	*Erythrobacteraceae*	0 (0)	0 (0)	0 (0)	0 (0)	0 (0)	0 (0)	0.05 (0.03)	0.38 (0.31)	0 (0)	0 (0)
Proteobacteria	*Orbaceae*	0 (0)	0.04 (0.03)	0 (0)	0 (0)	0 (0)	0.01 (0.01)	0.03 (0.02)	0.02 (0.02)	0.05 (0.05)	0.04 (0.1)
Proteobacteria	*Pasteurellaceae*	0 (0)	0 (0)	0 (0)	0 (0)	0.02 (0.02)	0.02 (0.01)	0.09 (0.06)	0.06 (0.04)	0.19 (0.18)	0.32 (0.18)
Proteobacteria	*Rhodobacteraceae*	0 (0)	0 (0)	0 (0)	0 (0)	0 (0)	0.58 (0.37)	0.18 (0.12)	0.32 (0.22)	0 (0)	0 (0)
Proteobacteria	*Rickettsiaceae*	0 (0)	0 (0)	0 (0)	0 (0)	0.1 (0.07)	0.03 (0.02)	0.01 (0.01)	0.69 (0.45)	0.3 (0.15)	1.14 (0.54)
Tenericutes	*Spiroplasmataceae*	1.54 (0.01)	1.02 (<0.01)	0 (0)	0 (0)	2.72 (0.03)	2.13 (0.02)	2.1 (0.02)	1.61 (<0.01)	0.72 (0.01)	0.35 (<0.01)
Unclassified	Unclassified	0.61 (0.29)	1.04 (0.41)	0.5 (<0.01)	1.89 (0.85)	1.64 (0.45)	1.37 (0.81)	1.24 (0.74)	1.1 (0.45)	1.59 (0.74)	1.2 (0.89)

Supplementary Table 5. Descriptive statistics and Wilcoxon–Mann–Whitney (WMW) test results based on the presence/absence of Microsporidia and *Spiroplasma* sp. PL03 reads. Statistical significance results are marked with a grey background.

Species	Infection	n	Mann–Whitney U test		
			Z	p(>|Z|)	r
Ae. vexans	Yes	13	-2.54	0.01	0.58
	No	6			
C. richiardii	Yes	9	-0.88	1	0.22
	No	7			
O. annulipes	Yes	34	-1.65	0.1	0.21
	No	29			
O. cantans	Yes	44	-3.02	0.002	0.34
	No	33			
O. sticticus	Yes	8	-2.49	0.01	0.69
	No	5			

Supplementary Table 6. Spearman correlation coefficients (rho, ρ) for the relationship between Microsporidia–*Spiroplasma* sp. PL03 (M & S) and Microsporidia–*Weissella* cf. *viridescens* (M & W) reads.

Species	Spearman's rank			
	M & S		M & W	
	rho	*p*	rho	*p*
Ae. vexans	0.13	0.685	0.81	< 0.001
C. richiardii	0.27	0.665	-	-
O. annulipes	0.18	0.299	0.45	0.006
O. cantans	0.1	0.517	0.58	< 0.001
O. sticticus	0.1	0.845	-	-
p	0.216		< 0.001	

Supplementary Table 7. Descriptive statistics and Wilcoxon–Mann–Whitney (WMW) results based on the number of *Spiroplasma* sp. PL03 reads in mosquitoes infected by Microsporidia (M) or both Microsporidia and *Weissella* cf. *viridescens* (M & W). Statistical significance results are marked with a grey background.

Species	Infection	n	Mann–Whitney U test		
			Z	p(>|Z|)	r
Ae. vexans	M	9	0	1	0
	M & W	4			
O. annulipes	M	30	-2.51	0.007	0.43
	M & W	4			
O. cantans	M	37	-0.34	0.748	0.05
	M & W	7			

Supplementary Table 8. The average weighted NSTI scores in mosquitoes infected and non-infected by Microsporidia.

Species	Infection	NSTI value (SD)
Ae. vexans	Yes	0.02 (0.01)
	No	0.02 (0.01)
C. richiardii	Yes	0.03 (0.02)
	No	0.04 (0.03)
O. annulipes	Yes	0.05 (0.04)
	No	0.07 (0.05)
O. cantans	Yes	0.06 (0.05)
	No	0.03 (0.02)
O. sticticus	Yes	0.04 (0.03)
	No	0.02 (0.01)

Supplementary Table 9. Mean relative abundances of the predicted pathways of the metagenome related to KEGG at level 2 among infected and non-infected mosquitoes.

Pathway Level 1	Pathway Level 2	*Ae. vexans*		*C. richiardii*		*O. annulipes*		*O. cantans*		*O. sticticus*	
		**Infected (SD)**	**Non-infected (SD)**	**Infected (SD)**	**Non-infected (SD)**	**Infected (SD)**	**Non-infected (SD)**	**Infected (SD)**	**Non-infected (SD)**	**Infected (SD)**	**Non-infected (SD)**
Cellular Processes	Cell growth and death	1.39 (0.21)	1.39 (0.17)	2.08 (0.42)	1.73 (0.55)	1.55 (0.29)	1.4 (0.17)	1.49 (0.14)	1.46 (0.37)	1.41 (0.14)	1.25 (0.19)
Cellular Processes	Cell motility	2.3 (1.24)	3.62 (0.58)	1.22 (0.68)	2.13 (0.85)	1.22 (1.17)	3.48 (0.78)	1.61 (1.26)	3.08 (1.11)	2.63 (1.1)	3.62 (0.71)
Cellular Processes	Cellular community - prokaryotes	0.18 (0.08)	0.2 (0.07)	0.09 (0.03)	0.15 (0.11)	0.14 (0.06)	0.22 (0.08)	0.15 (0.06)	0.19 (0.08)	0.23 (0.07)	0.26 (0.11)
Cellular Processes	Transport and catabolism	0.3 (0.09)	0.37 (0.04)	0.3 (0.1)	0.35 (0.06)	0.2 (0.11)	0.35 (0.06)	0.24 (0.12)	0.34 (0.06)	0.25 (0.09)	0.33 (0.04)
Environmental Information Processing	Membrane transport	2.53 (0.94)	2.34 (0.53)	2.56 (0.48)	2.48 (0.91)	2.74 (0.66)	2.41 (0.69)	2.52 (0.7)	2.39 (0.64)	2.77 (0.59)	2.9 (0.83)
Environmental Information Processing	Signal transduction	0.54 (0.17)	0.63 (0.07)	0.33 (0.06)	0.45 (0.16)	0.36 (0.13)	0.61 (0.12)	0.39 (0.14)	0.57 (0.16)	0.57 (0.12)	0.7 (0.15)
Environmental Information Processing	Signaling molecules and interaction	0 (0)	<0.01 (<0.01)	0 (0)	0 (0)	<0.01 (<0.01)	<0.01 (<0.01)	<0.01 (<0.01)	<0.01 (<0.01)	0 (0)	0 (0)
Genetic Information Processing	Folding, sorting and degradation	3.1 (0.21)	2.87 (0.09)	3.73 (0.46)	3.41 (0.62)	3.18 (0.33)	2.99 (0.13)	3.1 (0.26)	3.07 (0.4)	3.1 (0.31)	3.04 (0.12)
Genetic Information Processing	Replication and repair	5.19 (1.17)	4.74 (0.26)	7.15 (0.93)	5.84 (1.18)	6.92 (1.49)	5.09 (0.39)	6.35 (1.47)	5.14 (0.93)	5.67 (0.83)	4.68 (0.16)
Genetic Information Processing	Transcription	0.72 (0.14)	0.7 (0.06)	0.83 (0.08)	0.77 (0.12)	0.77 (0.09)	0.7 (0.06)	0.76 (0.1)	0.71 (0.1)	0.64 (0.08)	0.6 (0.06)
Genetic Information Processing	Translation	2.69 (0.68)	2.31 (0.16)	4.12 (0.65)	3.14 (0.97)	3.72 (0.92)	2.5 (0.25)	3.36 (0.89)	2.62 (0.71)	2.94 (0.5)	2.3 (0.12)
Human Diseases	Cancer: overview	0 (0)	0 (0)	0 (0)	0 (0)	0 (0)	0 (0)	0 (0)	<0.01 (0.01)	0 (0)	0 (0)
Human Diseases	Cardiovascular disease	0 (0)	<0.01 (0.01)	<0.01 (<0.01)	<0.01 (<0.01)	<0.01 (<0.01)	<0.01 (<0.01)	<0.01 (<0.01)	<0.01 (<0.01)	0 (0)	<0.01 (<0.01)
Human Diseases	Immune disease	<0.01 (<0.01)	<0.01 (<0.01)	<0.01 (<0.01)	<0.01 (<0.01)	<0.01 (<0.01)	<0.01 (<0.01)	<0.01 (<0.01)	<0.01 (<0.01)	<0.01 (<0.01)	<0.01 (<0.01)
Human Diseases	Infectious disease: bacterial	0.15 (0.09)	0.13 (0.05)	0.09 (0.05)	0.07 (0.07)	0.2 (0.12)	0.15 (0.08)	0.16 (0.06)	0.14 (0.08)	0.25 (0.09)	0.16 (0.05)

Pathway Level 1	Pathway Level 2	*Ae. vexans*		*C. richiardii*		*O. annulipes*		*O. cantans*		*O. sticticus*	
		Infected (SD)	Non-infected (SD)	Infected (SD)	Non-infected (SD)	Infected (SD)	Non-infected (SD)	Infected (SD)	Non-infected (SD)	Infected (SD)	Non-infected (SD)
Human Diseases	Infectious disease: parasitic	0.05 (0.03)	0.08 (0.01)	0.03 (0.02)	0.03 (0.01)	0.02 (0.03)	0.08 (0.02)	0.03 (0.02)	0.07 (0.03)	0.05 (0.03)	0.08 (0.01)
Human Diseases	Neurodegenerative disease	0.02 (0.03)	0.03 (0.05)	0.07 (0.1)	0.05 (0.08)	0.02 (0.05)	0.02 (0.04)	0.01 (0.02)	0.04 (0.06)	0.05 (0.13)	0.02 (0.04)
Metabolism	Amino acid metabolism	12.52 (1.35)	12.38 (0.29)	11.22 (1.12)	12.43 (1.23)	11.12 (1.38)	12.45 (0.56)	11.52 (1.34)	12.16 (0.93)	11.97 (0.78)	11.92 (0.78)
Metabolism	Biosynthesis of other secondary metabolites	2.59 (0.57)	2.29 (0.17)	1.87 (0.42)	2.19 (0.6)	2.19 (0.43)	2.32 (0.18)	2.28 (0.36)	2.18 (0.36)	2.33 (0.22)	2.31 (0.3)
Metabolism	Carbohydrate metabolism	14.16 (1.39)	13.42 (0.44)	12.33 (0.77)	13.19 (1.4)	13.86 (0.8)	13.59 (0.76)	13.89 (0.75)	13.44 (0.88)	14.36 (0.58)	14.13 (1.06)
Metabolism	Energy metabolism	5.13 (0.35)	5.4 (0.16)	5.55 (0.58)	5.27 (0.12)	4.96 (0.49)	5.3 (0.23)	5.1 (0.5)	5.43 (0.51)	5.21 (0.32)	5.07 (0.29)
Metabolism	Glycan biosynthesis and metabolism	3 (0.29)	2.74 (0.24)	3.04 (0.24)	3.1 (0.09)	3.03 (0.27)	2.92 (0.35)	3.17 (0.54)	2.93 (0.39)	2.98 (0.23)	2.82 (0.18)
Metabolism	Lipid metabolism	7.06 (1.03)	6.94 (0.47)	6.42 (1.26)	5.79 (0.82)	7.8 (1.55)	6.62 (1.01)	7.41 (1.48)	6.5 (0.95)	6.01 (0.81)	6.52 (1.3)
Metabolism	Metabolism of terpenoids and polyketides	11.66 (1.37)	11.87 (0.2)	13.15 (2.21)	13.55 (1.49)	10.44 (2.2)	12.09 (0.58)	10.93 (1.94)	12.45 (1.11)	13.42 (0.97)	12.6 (0.66)
Metabolism	Metabolism of other amino acids	8.53 (0.66)	8.19 (0.35)	8.58 (0.59)	8.98 (0.87)	8.87 (0.69)	8.29 (0.41)	8.79 (0.65)	8.48 (0.64)	9.53 (1.32)	8.55 (0.66)
Metabolism	Metabolism of cofactors and vitamins	8.22 (1.64)	7.93 (1.06)	7.99 (1.59)	7.43 (0.83)	9.68 (1.99)	7.59 (0.79)	9.52 (1.81)	7.81 (1.02)	6.78 (0.77)	7.11 (0.56)
Metabolism	Nucleotide metabolism	1.78 (0.37)	1.56 (0.08)	2.25 (0.29)	1.89 (0.31)	2.32 (0.48)	1.7 (0.13)	2.14 (0.49)	1.71 (0.27)	2.02 (0.37)	1.62 (0.09)
Metabolism	Xenobiotics biodegradation and metabolism	5.83 (1.91)	7.51 (0.78)	4.64 (0.85)	5.25 (1.65)	4.37 (2.04)	6.81 (0.97)	4.74 (1.86)	6.71 (1.39)	4.53 (1.88)	7.02 (1.96)
Organismal System	Immune system	0.05 (0.05)	0.03 (0.02)	0.06 (0.05)	0.02 (0.03)	0.03 (0.04)	0.03 (0.02)	0.03 (0.03)	0.04 (0.03)	0.02 (0.02)	0.02 (0.02)
Organismal Systems	Development and regeneration	0 (0)	0 (0)	<0.01 (<0.01)	0 (0)	0 (0)	<0.01 (<0.01)	<0.01 (<0.01)	<0.01 (<0.01)	<0.01 (<0.01)	<0.01 (<0.01)
Organismal Systems	Digestive system	0.02 (0.02)	0.02 (0.01)	0.02 (0.01)	0.02 (0.02)	0.01 (0.02)	0.02 (0.02)	0.02 (0.02)	0.02 (0.01)	0.01 (0.01)	0.01 (0.01)
Organismal Systems	Endocrine system	0.07 (0.04)	0.08 (0.02)	0.05 (0.02)	0.06 (0.02)	0.05 (0.04)	0.07 (0.03)	0.05 (0.03)	0.08 (0.03)	0.05 (0.04)	0.09 (0.03)
Organismal Systems	Environmental adaptation	0.23 (0.07)	0.21 (0.02)	0.25 (0.04)	0.23 (0.06)	0.24 (0.05)	0.21 (0.03)	0.23 (0.05)	0.22 (0.05)	0.23 (0.07)	0.27 (0.05)

Supplementary Table 10. *Spiroplasma* species isolated from mosquitoes. Asterisks indicate the same species.

***Spiroplasma* species**	**Mosquito species**	**Study area**	**Reference**
S. culicicola	*Aedes sollicitans*	New Jersey, USA	[1]
S. sabaudiense	*Aedes sticticus*	Chamousset, France	[2]
S. sabaudiense	*Aedes vexans*	Chamousset, France	[2]
Spiroplasma sp.*	*Anopheles coluzzii*	Vallée du Kou, Burkina Faso	[3]
Spiroplasma sp.*	*Anopheles gambiae*	Soumousso, Burkina Faso	[3]
Spiroplasma sp.	*Anopheles funestus*	Lwanda, Kenya	[4]
Spiroplasma sp.**	*Anopheles gambiae*	Kirindo, Kenya	[5]
Spiroplasma sp.**	*Anopheles gambiae*	Mageta Island, Kenya	[5]
Spiroplasma sp.**	*Anopheles gambiae*	Mwea, Kenya	[5]
S. taiwanense	*Culex tritaeniorhynchus*	Taishan, Taiwan	[5]
S. diminutum	*Culex annulus*	Taishan, Taiwan	[6]
S. diminutum	*Culex tritaeniorhynchus*	Taishan, Taiwan	[7]

Supplementary Bibliography

1. Hung SHY, Chen T., Whitcomb R., et al (1987) Spiroplasma culicicola sp. nov. from the Salt Marsh Mosquito Aedes sollicitans. Int J Syst Evol Microbiol 37:365–370. https://doi.org/https://doi.org/10.1099/00207713-37-4-365
2. Abalain-Colloc ML, Chastel C, Tully JG, et al (1987) Spiroplasma sabaudiense sp. nov. from Mosquitoes Collected in France. Int J Syst Evol Microbiol 37:260–265
3. Segata N, Baldini F, Pompon J, et al (2016) The reproductive tracts of two malaria vectors are populated by a core microbiome and by gender-and swarm-enriched microbial biomarkers. Sci Rep 6:1–10. https://doi.org/10.1038/srep24207
4. Lindh JM, Terenius O, Faye I (2005) 16S rRNA Gene-Based Identification of Midgut Bacteria from Field-Caught. Appl Environ Microbiol 71:7217–7223. https://doi.org/10.1128/AEM.71.11.7217
5. Chepkemoi ST, Mararo E, Butungi H, et al (2017) Identification of Spiroplasma insolitum symbionts in Anopheles gambiae. Wellcome Open Res 2:90. https://doi.org/10.12688/wellcomeopenres.12468.1
6. Abalain-Colloc ML, Rosen L, Tully JG, et al (1988) Spiroplasma taiwanense sp. nov. from Culex tritaeniorhynchus Mosquitoes Collected in Taiwan. Int J Syst Evol Microbiol 38:103–107
7. Williamson DL, Tully JG, Rosen L, et al (1996) Spiroplasma diminutum sp. nov., from Culex annulus Mosquitoes Collected in Taiwan. Int J Syst Evol Microbiol 46:229–233
